# Targeting NCAPG with Arundanine Suppresses Neutrophil/NET-Mediated Immune Evasion and Enhances CD8^+^ T Cell Function in Lung Squamous Cell Carcinoma

**DOI:** 10.7150/ijbs.134668

**Published:** 2026-09-11

**Authors:** Xuyu Gu, Dongning Lu, Ming Deng, Tao Ge, Kaiqi Jin, Xinnan Xu, Li Xu

**Affiliations:** 1 Department of Oncology, Shanghai Pulmonary Hospital, School of Medicine, Tongji University, Shanghai, 200433, China.; 2 Department of Thoracic Surgery, Shanghai Pulmonary Hospital, School of Medicine, Tongji University, Shanghai, 200433, China.

**Keywords:** NCAPG, neutrophils, CD8^+^ T cells, LUSC, Arundanine

## Abstract

Non-SMC condensin I complex subunit G (NCAPG) is implicated in tumor progression and TGF-β pathway activation, yet its roles in oncogenesis and immune modulation within the lung squamous cell carcinoma (LUSC) microenvironment remain largely unexplored. Here, we used bioinformatics analysis to identify key upregulated genes in LUSC and assessed the effects of NCAPG and its upstream regulator E2F transcription factor 8 (E2F8) on malignant phenotypes via gain- and loss-of-function assays in NCI-H226 and NCI-H1703 LUSC cell lines, with in vivo efficacy validated using KLN205 cell isografts and NTCU-induced primary LUSC mouse models. Neutrophil polarization, neutrophil extracellular trap (NET) formation, and CD8⁺ T cell cytotoxicity was analyzed in co-culture systems and tumor tissues. Arundanine was identified as a potential NCAPG inhibitor via molecular docking and tested as monotherapy or in combination with anti-PD-1 therapy. We found that NCAPG was significantly upregulated in LUSC, and NCAPG depletion markedly impaired LUSC cell proliferation, migration, and invasion while promoting apoptosis. Mechanistically, NCAPG activated TGF-β signaling, which drove pro-tumor N2 neutrophil polarization and induced NETosis; these NETs upregulated PD-L1 expression on tumor cells and suppressed the expansion and cytotoxic function of CD8⁺ T cells. E2F8 was found to transcriptionally activate NCAPG by directly binding to its promoter. Furthermore, Arundanine destabilized NCAPG protein, suppressed TGF-β-mediated NET formation, and potentiated anti-PD-1 therapy to significantly prolong survival in LUSC models. Collectively, the E2F8/NCAPG axis promotes LUSC progression by orchestrating a TGF-β-driven immunosuppressive microenvironment, and targeting NCAPG with Arundanine represents a promising therapeutic strategy to overcome immune evasion and sensitize LUSC to PD-1 blockade.

## Introduction

Lung cancer remains the leading cause of cancer-related morbidity and mortality worldwide, with nearly 2.5 million new cases and more than 1.8 million deaths reported in 2022 1. Lung squamous cell carcinoma (LUSC) constitutes approximately 25-30% of all non-small cell lung cancer (NSCLC) and represents a clinically distinct subtype with strong links to smoking-related genomic injury 2. For metastatic LUSC, platinum-based chemotherapy combined with immune checkpoint blockade has become a major first-line treatment strategy, as exemplified by the survival benefit of pembrolizumab plus chemotherapy in metastatic squamous NSCLC 3, 4. However, durable responses remain limited to a subset of patients, and primary or acquired resistance to PD-1/PD-L1 blockade remains a major clinical challenge 5. Compared with lung adenocarcinoma (LUAD), LUSC has fewer actionable driver alterations and limited effective targeted therapies, partly because recurrent alterations in LUSC often involve complex copy-number changes, tumor suppressor loss, and pathway-level dysregulation rather than single dominant kinase drivers 5-8. Therefore, identifying tumor-intrinsic regulators that drive LUSC progression and immune evasion may provide new therapeutic opportunities.

Our preliminary bioinformatics analyses identified Non-SMC Condensin I Complex Subunit G (NCAPG) as an aberrantly upregulated gene in LUSC. NCAPG is a core component of the condensin I complex, essential for chromosome condensation and stabilization during cell division 9. Aberrant activation of mitosis-related and chromosomal segregation programs is closely associated with uncontrolled proliferation, genomic instability, and aggressive tumor behavior 10. Emerging evidence implicates NCAPG in NSCLC progression, prognosis, and immune cell infiltration, supporting its potential relevance as both an oncogenic regulator and immune-associated biomarker 11, 12. Specifically, NCAPG has been shown to promote LUAD through stimulation of the transforming growth factor β (TGF-β) pathway 13. However, the specific roles and mechanisms of NCAPG in LUSC remain poorly defined.

Neutrophils constitute the most abundant type of circulating myeloid cells and exert substantial influence over the tumor microenvironment (TME) 14. Beyond their antimicrobial functions, these cells can initiate a specialized process known as NETosis, during which they expel neutrophil extracellular traps (NETs), which are extracellular networks composed of decondensed chromatin fibers decorated with various granular proteins, including myeloperoxidase (MPO) and citrullinated histone H3 (cit-H3) 15, 16. In cancer, NETs have been implicated in tumor growth, metastasis, dormant tumor-cell awakening, immune evasion, and therapy resistance 17, 18. Importantly, NETs can promote T-cell dysfunction through PD-L1-dependent mechanisms and can physically shield tumor cells from CD8⁺ T-cell and natural killer cell cytotoxicity 19, 20. Analogous to macrophages, tumor-associated neutrophils (TANs) exhibit plasticity, polarizing into either an anti-tumor N1 or a pro-tumor N2-like state, which are commonly associated with distinct functional and phenotypic markers (e.g., CD86, iNOS, CD95/Fas, and TNF-α for N1-like states; CD206, Arg-1, CD184, and VEGFA for N2-like states) 19, 21, 22. The immunosuppressive N2 phenotype is frequently driven by tumor-derived TGF-β 19, 21. Since NCAPG modulates TGF-β signaling in other contexts 13, we hypothesized that NCAPG drives LUSC progression by orchestrating TAN polarization. In this study, we identify E2F transcription factor 8 (E2F8) as a key upstream regulator of NCAPG and demonstrate that the E2F8/NCAPG axis promotes N2 neutrophil polarization and CD8^+^ T cell exhaustion. Furthermore, we screen and validate Arundanine as a potent inhibitor of this axis with therapeutic potential.

## Materials and Methods

### Cells

Human bronchial epithelial cells BEAS-2B (SNL-203) were sourced from SUNCELL Biotechnologies (Wuhan, Hubei, China). LUSC cell lines NCI-H226 (CL-0396), NCI-H1703 (CL-0390), and NCI-H520 (CL-0402) were obtained from Procell Life Science (China). These cells were routinely maintained in RPMI-1640 supplemented with 10% fetal bovine serum (FBS) along with 1% penicillin-streptomycin antibiotic mixture. Human peripheral blood neutrophils (IMP-H209) were purchased from Xiamen Immocell Biotechnology (China) and grown in human peripheral blood neutrophil-specific medium (CM-H197, Procell). All cultures were incubated under standard conditions of 37℃ with a humidified atmosphere containing 5% CO₂.

Lentiviral vectors for NCAPG knockdown, E2F8 knockdown, and NCAPG overexpression, along with corresponding negative control (NC) vectors were acquired from VectorBuilder Inc (Guangzhou, Guangdong, China). NCI-H226 and NCI-H1703 cells were incubated with lentivirus (titer 10^8^ TU/mL) and 8 μg/mL polybrene for 1 d, followed by medium replacement and further incubation for one additional day. Puromycin (2 μg/mL) was used to select transfected cells. To upregulate TGF-β, cells were exposed to 10 ng/mL TGF-β recombinant protein (HY-P70543, MedChemExpress, Monmouth Junction, NJ, USA) for 24 h, with the control group receiving an equivalent amount of PBS (pH 7.4, Gibco, Thermo Fisher Scientific).

For validation assays, frozen primary human peripheral blood neutrophils (Catalog #200-0384, STEMCELL Technologies, Vancouver, BC, Canada) were used. According to the supplier, these cells were isolated from peripheral blood by negative immunomagnetic separation and characterized as CD16^⁺^CD66b^⁺^ neutrophils. Cells were obtained under IRB-approved donor consent procedures by the supplier. After thawing following the supplier's instructions, neutrophils were washed, resuspended in neutrophil culture medium, and used immediately for conditioned-medium stimulation assays.

### Conditioned medium collection

LUSC cells were incubated in six-well plates at 2 × 10^5^ cells/well for 24 h. The conditioned medium (CM), was collected and used to culture neutrophils with 50% LUSC-CM for 12 h. After culture, the neutrophils were maintained in fresh medium alone for an additional 24 h. The supernatant was collected again for further analysis.

### Cell counting kit-8 (CCK-8) method

A CCK-8 assay kit (CA1210, Solarbio Science, China) was employed to assess cell viability. After different treatments, NCI-H226 and NCI-H1703 cells were plated in 96-well plates and cultured for 0, 12, 24, 36, and 48 h. At each designated time point, 10 μL of CCK-8 reagent was added to each well. The plates were then returned to the incubator for an additional 1 h at 37℃. The optical density (OD) was quantified at 450 nm utilizing a microplate reader (Multiskan FC, Thermo Fisher Scientific, Waltham, MA, USA).

### Colony formation assay

To assess long-term proliferative capacity, NCI-H226 and NCI-H1703 cells were plated in six-well plates at 1000 cells/well and incubated at 37 °C in a 5% CO₂ atmosphere for 2 weeks. After incubation, visible colonies were fixed with paraformaldehyde, followed by staining with 0.1% crystal violet solution. Colony counting was performed manually under an inverted light microscope (CKX53, Olympus, Tokyo, Japan), considering only those clusters comprising at least 50 individual cells as valid colonies.

### Wound healing assay

After overnight starvation, NCI-H226 and NCI-H1703 cells were seeded into six-well plates and cultured until reaching approximately 90% confluency, forming a uniform monolayer. A scratch was created using a 200 μL pipette tip. Loose debris was removed by gentle washing with PBS, after which the cultures were switched to serum-free medium to sustain migration without confounding growth stimulation. Photographic documentation of the wound area was obtained immediately after scratching (0 h) and again after 24 h using an inverted microscope equipped with a digital camera. The extent of wound closure was quantitatively assessed by comparing the gap width across time points.

### Transwell assays

To evaluate invasive behavior, a Matrigel-coated Transwell system with 8 μm pore membranes was used for NCI-H226 and NCI-H1703 cells. The upper surface of the inserts was pre-coated with diluted Matrigel to mimic extracellular matrix barriers. Cells were harvested, resuspended in serum-free medium, and introduced into the upper compartment at 5 × 10^4^ cells/well. The lower chamber was filled with complete medium to serve as a chemoattractant. After 24 h, cells that successfully traversed the membrane and Matrigel layer to the underside were fixed and stained with 0.1% crystal violet for 30 min. Invasive cells were enumerated under the microscope (BX53, Olympus).

### TUNEL assays

A TUNEL apoptosis detection kit (40308ES20, YEASEN Biotechnology Co. Ltd.) was used to assess apoptosis in NCI-H226 and NCI-H1703 cells, as well as in mouse tumor tissues (details provided later). Cells were fixed and then incubated with 0.2% Triton X-100 to permeabilize the membrane. Tumor tissue sections were deparaffinized using xylene and rehydrated with a gradient ethanol series, followed by incubation with Proteinase K solution at 20-25 °C for 20 min. After equilibration with buffer for 30 min, TdT incubation buffer was added, and samples were incubated in the dark. The samples were counterstained with DAPI and mounted with antifade medium. The TUNEL-positive staining rate was then calculated.

### Flow cytometry

Cultured TANs were collected, washed with PBS, and resuspended. Cells were incubated with FITC-CD95 (11-0959-42, Thermo Fisher Scientific) and PE-CD184 (12-9999-42, Thermo Fisher Scientific) antibodies were loaded for incubation at 4 °C for 30 min in the dark. After removing excess primary antibodies, the cell suspensions were subjected to fluorescence-activated cell sorting on a BD FACS Aria II flow cytometer (BD Biosciences), with FlowJo software applied for data analysis.

### Animal experiments

All procedures involving animal experiments were approved by the Institutional Animal Ethics Committee (Approval number: K25-392Y). DBA/2 mice were obtained from SLAC Laboratory (China) and housed in an SPF facility with a temperature range of 20-25 °C, relative humidity < 70%, and a 12-h light/dark cycle. The mice were provided unrestricted access to standard laboratory rodent chow and autoclaved drinking water. The mouse lung cancer cell line KLN205 (CL-0140), obtained from Procell, was maintained in DMEM containing 10% FBS and 1% antibiotics. Stable E2F8 knockdown or NCAPG overexpression in KLN205 cells was achieved using lentiviral vectors.

To establish animal models with subcutaneous tumors, approximately 2 × 10^6^ KLN205 cells were implanted into the DBA/2 mice subcutaneously. Tumor volume was examined every 5 d with calipers, using the formula 0.5 × length × width^2^. After 25 d, mice were sacrificed with an intraperitoneal injection of pentobarbital sodium (150 mg/kg). Tumor tissues were excised, weighed, and either embedded in paraffin or stored at low temperatures for further analysis. Additional cohorts of DBA/2 mice were administered transfected KLN205 cells via the tail vein to analyze tumor cell metastasis or to monitor their survival within 60 d.

For *in vivo* therapeutic evaluation, Arundanine (Cat. No. HY-N11803, MedChemExpress) was dissolved in DMSO to prepare a high-concentration stock and subsequently formulated in a sterile vehicle comprising physiological saline with 5% DMSO and 2% Tween-80. Starting on day 8 post-tumor inoculation, Arundanine was administered at dosages of 5 or 10 mg/kg via intraperitoneal (i.p.) injection. The stock solution in 100% DMSO was first mixed with an equal volume of Tween-80, followed by slow dropwise addition of saline with continuous vortexing. The final dosing solutions remained clear at the required concentrations, up to 10 mg/mL. All formulations were prepared fresh daily and administered within 30 min. Control mice received an equivalent volume of the vehicle solution alone.

For the* in vivo* CD8⁺ T cell depletion experiments, mice were administered 200 μg of an anti-mouse CD8α monoclonal antibody (Clone 53-6.7) via intraperitoneal (i.p.) injection. The treatment was initiated on day 8 post-tumor inoculation and repeated every 3 days. The every-3-day dosing interval was selected based on established *in vivo* CD8⁺ T-cell depletion protocols and previous tumor-immunology studies using repeated anti-CD8 antibody administration at 3- to 4-day intervals to maintain CD8⁺ T-cell depletion during the experimental period 23, 24.

For the in vivo TGF-β1 rescue experiments, mice bearing NCAPG-knockdown KLN205 tumors were administered recombinant TGF-β1 protein starting on day 8 after tumor inoculation. Recombinant TGF-β1 was reconstituted according to the manufacturer's instructions and diluted in sterile PBS containing 0.1% BSA as a carrier protein. Mice received recombinant TGF-β1 at 0.5 μg per mouse by intraperitoneal injection every 2 days until the experimental endpoint. Control cohorts were administered an equivalent volume of the 0.1% BSA vehicle solution.

### Enzyme-linked immunosorbent assay (ELISA)

The contents of tumor necrosis factor α (TNF-α), interleukin-8 (IL-8), and vascular endothelial growth factor A (VEGFA) in TAN supernatant and tumor tissue homogenates were quantified using ELISA kits for Human IL-8 (E-EL-H6008, Elabscience Biotechnology Co., Ltd.), Human TNF-α (E-EL-H0109, Elabscience), Human VEGFA (E-EL-H0111, Elabscience), Mouse KC (IL-8, MBS8579382, Mybiosource, San Diego, CA, USA), and Mouse VEGFA (E-EL-M1292, Mybiosource). The supernatant was centrifuged at 1,000 × g for 30 min, and tumor tissue homogenates were prepared with a glass homogenizer on ice, followed by centrifugation at 5,000 × g. The samples were then added to microplates and incubated at 37 °C for 90 min. After incubation with a biotinylated antibody, OD at 450 nm was measured, and cytokine levels were determined using a standard curve.

### Western blot (WB) analysis

Cells or mouse tumor tissue samples were lysed in RIPA buffer to extract supernatant. Protein content was quantified using a BCA assay, and 20 µg of each sample was separated by 12% SDS-PAGE gel before electrophoretic transfer to PVDF membranes. After blocking, the membranes were probed at 4 °C overnight with primary antibodies against NCAPG (1:1000, 24563-1-AP, Proteintech Group, Inc.), Ki67 (1:1000, ab16667, Abcam Inc), PCNA (1:5000, 10205-2-AP, Proteintech), p-SMAD2 (1:1000, ab280888, Abcam), p-SMAD3 (1:2000, ab52903, Abcam), Arg-1 (1:5000, PA5-29645, Thermo Fisher Scientific), iNOS (1:200, PA1-036, Thermo Fisher Scientific), E2F8 (1:500, 13425-1-AP, Proteintech) and GAPDH (1:30000, 30202ES40, YEASEN). After washing, the membranes were exposed to HRP-conjugated secondary antibodies and developed using enhanced chemiluminescence.

### Reverse transcription-quantitative polymerase chain reaction (RT-qPCR)

Total RNA was isolated from LUSC cells utilizing Beyozol reagent (R0011, Beyotime Biotechnology, China) and reverse-transcribed to cDNA. Then, qPCR was conducted utilizing qPCR SYBR Green Master Mix (11184ES03, YEASEN) on a 7500 Real-Time PCR system. Gene expression levels were normalized to GAPDH and calculated by the 2^-ΔΔCt^ method. Primer sequences used were as follows: NCAPG (F) GGAACTGGCATTTGACACGAGC, NCAPG (R) GCTGCTCTAACAATGGGTGGCT; E2F8 (F) GAGGCTCAAAGAGGGCAAGCAT, E2F8 (R) ATGAGCACTGCGTGAGAGGGAT; GAPDH (F) GTCTCCTCTGACTTCAACAGCG, GAPDH (R) ACCACCCTGTTGCTGTAGCCAA.

### Chromatin immunoprecipitation (ChIP)-qPCR

Following the guidelines of the Sonication ChIP Kit (RK20258, ABclonal Biotechnology, China), NCI-H226 and NCI-H1703 cells were fixed in 1% formaldehyde for 15 min to cross-link proteins to DNA, with the reaction quenched by the addition of glycine. Cell pellets were lysed, and ChIP sonication buffer was added. Samples were sonicated and immunoprecipitated with E2F8 antibody (1:50, 13425-1-AP, Proteintech) and rabbit IgG control at 4 °C for 4 h. DNA was then purified, and qPCR was performed using primers specific to the NCAPG promoter.

### Luciferase assay

The NCAPG promoter sequence was retrieved from the UCSC database (https://genome.ucsc.edu/index.html) 25 and inserted into pGL3. Cells were plated in 96-well plates, and the pGL3 vector and Renilla luciferase plasmid were transfected into E2F8-knockdown cells utilizing Lipofectamine 3000. Luciferase activity in cells was evaluated 48 h later employing the luciferase reporter system.

### Immunofluorescence staining

Mouse tumor tissue paraffin sections were dewaxed and then rehydrated in ethanol. Antigen retrieval was carried out by immersing the sections in citrate buffer and heating them in a microwave. After blocking with 10% goat serum for 1 h, the sections were incubated overnight at 4 °C with primary antibodies, including rabbit anti-iNOS (1:20, PA1-036, Thermo Fisher), rabbit anti-CD206 (1:50, PA5-101657, Thermo Fisher), and rat anti-Ly6G (1:50, 14-5931-82, Thermo Fisher). After removing excess primary antibody, the sections were exposed to Alexa Fluor 647-conjugated (1:200, ab150083, Abcam) and Alexa Fluor 488-conjugated (ab150165, Abcam) IgG in the dark for 1 h. Nuclei were counterstained using DAPI solution, after which the slides were coverslipped and mounted. Double-positive cells were counted under the fluorescence microscope (BX53, Olympus, Tokyo, Japan).

### Immunohistochemistry (IHC)

Paraffin-embedded mouse tumor tissue sections were dewaxed and rehydrated. Following antigen retrieval in citrate buffer and blocking, the sections were probed overnight at 4 °C with antibodies against E2F8 (1:100, PA5-106867, Thermo Fisher) and NCAPG (1:20, 24563-1-AP, Proteintech). After washing, HRP-conjugated IgG (1:200, GTX213110-01, GeneTex) was applied for 2 h. Nuclear counterstaining was achieved using hematoxylin solution, and the slides were dehydrated, cleared, and coverslipped with neutral resin for microscopic observation.

### Generation of primary LUSC in mice

To generate a primary LUSC model, BALB/c (SLAC Laboratory) mice were acclimated in SPF-grade facilities for a week. N-nitroso-tris-chloroethylurea (NTCU, 40 mM, Cat. No.: HY-W887123, MedchemExpress) was dissolved in acetone and topically applied to the shaved dorsal skin of the mice twice weekly for 30 weeks. Starting from week 30, the mice were allocated into 3 groups, receiving NCAPG-specific small interfering (si) RNA (10 nM/mouse), E2F8-specific siRNA (10 nM/mouse), or negative control siRNA (scrambled siRNA, 10 nM/mouse), each consisting of 6-8 mice. The siRNA was administered via tail vein injection with a cationic lipid transfection reagent (Invivofectamine 3.0, Thermo Fisher Scientific), twice a week for 4 weeks. After week 34, the mice were anesthetized and euthanized, and lung tissues were harvested for subsequent analysis.

### Histological examination and tumor nodule counting in mouse lung tissues

The collected lung tissues were washed with PBS, and images were captured under a macroscope to record tumor appearance and distribution. Subsequently, the lung tissues were fixed, dehydrated, paraffin-embedded, and cut into 4-μm sections. The formation of tumor nodules was analyzed using Hematoxylin and eosin (HE) staining. Two experienced pathologists, blinded to the group information, independently counted the total number of lung tumor nodules. If the difference exceeded 10%, a third evaluator determined the final count.

### Assessment of N2 TANs in primary mouse LUSC tumors

Fresh lung tissues from mice were digested for 30 min. The digest was then passed through a 70 μm nylon mesh strainer to remove debris. Remaining erythrocytes were eliminated by a 5-min lysis step. The cells were resuspended in PBS and incubated with a mixture of anti-CD11b-APC (#101212), anti-Ly6G-PE (#127608), and anti-CXCR4/CD184-FITC (#146505) (all provided by BioLegend) at the recommended concentrations for 30 min on ice, avoiding light exposure. After two washes with FACS buffer, samples were acquired on a BD FACSCanto II flow cytometer. Data analysis was conducted with FlowJo software (v10) to determine the population of CD11b^+^Ly6G^+^CD184^+^ (N2 type) TANs.

### Immunofluorescence staining for NET detection

Following routine deparaffinization, rehydration, antigen retrieval, permeabilization, and 5% BSA washing, the prepared tissue sections were exposed to anti-cit-H3 (ab5103, 1:200, Abcam) and anti-MPO (ab9535, 1:200, Abcam) overnight at 4 °C. The next day, Alexa Fluor 488/594-labeled IgG (1:500, Thermo Fisher) was added and incubated at 20-25℃ for 1 h. After DAPI staining of the nuclei, the slides were mounted. Images were taken under a Zeiss LSM880 confocal microscope, and ImageJ 1.53t was applied to analyze the fluorescence intensity of the cit-H3 and MPO double-positive regions as an indicator of NET formation.

### Protein degradation and ubiquitination assays

To assess the degradation pathway, NCI-H226 and NCI-H1703 cells were pre-treated with 10 μM MG132 (proteasome inhibitor) or 50 μM Chloroquine (lysosome inhibitor) for 2 h prior to the addition of Arundanine. For the ubiquitination assay, cells treated with Arundanine and MG132 were lysed in RIPA buffer. Equal amounts of protein were incubated with an anti-NCAPG antibody overnight at 4 °C, followed by precipitation with Protein A/G magnetic beads. The immunoprecipitants were washed, boiled in SDS loading buffer, and subjected to Western blot analysis using an anti-ubiquitin antibody.

### Cellular Thermal Shift Assay (CETSA)

To evaluate direct drug-target engagement, NCI-H226 and NCI-H1703 cells were treated with Arundanine or a DMSO vehicle control for 2 h. Cells were harvested, washed, and aliquoted equally into PCR tubes. The aliquots were subjected to a temperature gradient (40 °C to 60 °C) for 3 min using a thermal cycler, followed by immediate cooling at room temperature for 3 min. Cells were lysed via repeated freeze-thaw cycles in liquid nitrogen, and the soluble protein fractions were isolated by high-speed centrifugation (20,000 × *g*, 20 min). The remaining soluble NCAPG protein at each temperature was quantified via Western blot analysis.

### Tissue microarray (TMA) analysis

LUSC TMAs containing 87 paired LUSC and adjacent tumor tissue samples and the accompanying clinical characteristics (including sex, TNM staging, and treatment histories) were procured from Shanghai Outdo Biotech (Shanghai, China). All samples were de-identified, and the authors had no access to identifiable patient information. According to the documentation provided by the supplier, the specimens were collected in accordance with applicable ethical requirements and donor consent procedures. The use of these de-identified commercial samples was reviewed by the Institutional Ethics Committee, which waived the requirement for additional informed consent.

The expression of E2F8, NCAPG, PD-L1, and MPO in tissue samples was analyzed using IHC. Based on the median staining intensity of E2F8 or NCAPG, patients were allocated into high-expression and low-expression cohorts, and differences in PD-L1 and MPO expression were compared. Additionally, clinical data were collected from 17 patients who received anti-PD-1 monoclonal antibody treatment among the 87 cases. Based on treatment response (according to RECIST criteria), patients were categorized into the responder (n = 10) and non-responder (n = 7) cohorts. The expression of E2F8 and NCAPG in these two groups of samples was compared.

### Statistical analysis

All statistical analyses were conducted employing GraphPad Prism 10.3.1. Results are expressed as the mean ± SD. One-way or two-way analysis of variance was used to compare differences across multiple groups, with Tukey's test for post hoc comparison. A *p*-value of < 0.05 was considered statistically significant.

## Results

### NCAPG is significantly upregulated in LUSC and correlates with tumor malignancy

To identify potential therapeutic targets for LUSC, we analyzed differentially expressed genes (DEGs) in the GSE31552 dataset 26, and intersected them with the top 1,000 upregulated genes from GEPIA (http://gepia.cancer-pku.cn/detail.php?gene). This integrated analysis yielded 68 robustly upregulated candidate genes (Fig. S1A-B) 27. Among these genes, NCAPG was noted to induce stemness and tumor progression in LUAD by promoting glycolysis. Our database cross-validation revealed that NCAPG was markedly upregulated in LUSC tissues versus non-tumor counterparts (Fig. S1C, Supplementary Table 1). However, the role of NCAPG in LUSC remains unclear. We found higher NCAPG levels in LUSC cell lines than in normal BEAS-2B cells (Fig. S1D-E). To investigate its functional role, we established stable NCAPG-knockdown (NC^KD^) cell lines using lentiviral vectors, achieving efficient depletion in NCI-H226 and NCI-H1703 cells (Fig. S1F). NCAPG depletion substantially impaired LUSC cell proliferation, as evidenced by CCK-8 and colony formation assays (Fig. S1G-H), accompanied by downregulated proliferative markers Ki67 and PCNA (Fig. S1I). Furthermore, silencing NCAPG triggered significant cell apoptosis (Fig. S1J) and attenuated the migration and invasiveness of NCI-H226 and NCI-H1703 cells (Fig. S1K-L).

To validate these findings in vivo and assess localized tumor growth kinetics alongside the immediate immune microenvironment, we subcutaneously inoculated DBA/2 mice with NCAPG-deficient KLN205 cells. NC^KD^ tumors showed substantially slower growth kinetics and reduced mass compared to control tumors (Fig. 1A-C). Immunohistochemical (IHC) analysis revealed an increase in cleaved caspase-3 staining alongside a sharp reduction in PD-L1 expression in NC^KD^ tumor tissues (Fig. 1D-E). Strikingly, NCAPG deficiency remodeled the tumor immune microenvironment, marked by an enhanced infiltration of CD8⁺ T cells and an enrichment of TNF-α⁺ and GZMB⁺ cytotoxic subsets (Fig. 1F-H). These results suggest that NCAPG promotes LUSC growth by restraining CD8⁺ T cell-mediated anti-tumor immunity. To confirm that the NC^KD^-mediated growth inhibition was immune-dependent, we depleted CD8⁺ T cells in mice using anti-CD8 monoclonal antibodies. CD8 depletion almost completely abolished the tumor-suppressive effect of NCAPG silencing, as evidenced by restored tumor growth rates and diminished cleaved caspase-3 levels (Fig. 1I-M).

### NCAPG drives immunosuppressive N2 neutrophil polarization via the TGF-β signaling axis

Given the established link between NCAPG and TGF-β signaling in adenocarcinoma 13, we hypothesized that NCAPG modulates the immune landscape in LUSC. Indeed, bioinformatics supported a significant positive link between NCAPG expression and neutrophil infiltration (Fig. S2A) 28. To dissect the mechanism, we analyzed cytokine profiles in KLN205 isogeneic models. NCAPG depletion markedly reduced TGF-β1 concentrations in tumor tissues (Fig. 2A). However, the number of TGFBR1⁺ or TGFBR2⁺ CD8⁺ T cells showed no significant change (Fig. 2B). These results indicate that NCAPG-mediated release of TGF-β1 did not directly affect CD8 cells. To determine the role of NCAPG in regulating TGF-β1, we assessed its transcriptional activity. NCAPG knockdown in NCI-H226 and NCI-H1703 cells led to a significant reduction in TGFB1 mRNA levels (Fig. S2B). Furthermore, luciferase reporter assays confirmed that NCAPG depletion directly impaired the transcriptional activation of the TGF-β1 promoter (Fig. S2C). WB analysis confirmed that NCAPG silencing dampened downstream TGF-β signaling, evidenced by reduced phosphorylation of SMAD2 and SMAD3 (Fig. S2D). We next evaluated whether NCAPG-regulated secretomes influence neutrophil plasticity. Neutrophils cultured in CM from NC^KD^ cells exhibited a phenotypic shift from N2 to N1, characterized by decreased secretion of N2-associated cytokines (IL-8, VEGFA) and increased secretion of TNF-α (Fig. S2E-F). This phenotypic switch was corroborated by the downregulation of the N2 markers Arginase-1 (Arg-1) and CD184 and upregulation of the N1 markers iNOS and CD95 (Fig. S2G-H). Crucially, exogenous TGF-β supplementation reversed the NC^KD^-induced N1 polarization in vitro, restoring Arg-1 expression and CD184 high populations (Fig. S2I-J). To confirm the clinical relevance of these immune modulations and ensure they were not exclusive to the IMP-H209 immortalized cell line, we repeated the key CM co-culture assays using freshly isolated primary human peripheral blood neutrophils. Strikingly, primary human neutrophils exhibited identical phenotypic shifts: NCAPG-depleted LUSC CM significantly downregulated NETosis markers (Fig. S2K) and markedly abrogated NET formation compared to control LUSC CM.

To confirm that the reduction of TGF-β1 was a direct tumor-intrinsic effect rather than a secondary consequence of altered immune recruitment, we analyzed the cell-free culture supernatant of isolated LUSC cells. ELISA confirmed that NCAPG knockdown in NCI-H226 and NCI-H1703 cells significantly decreased the secretion of TGF-β1 into the CM (Fig. S2L). These data confirm that the NCAPG/TGF-β axis affects human neutrophil plasticity and effector function.

The numbers of CD45⁺CD11b⁺Ly6G⁺CD86⁺ (N1 TANs) and CD206⁺ (N2 TANs) cells were subsequently analyzed in mouse tumor tissues. A significant reduction in N2 TANs and a concomitant increase in N1 TANs was found in NC^KD^ tumors (Fig. 2C-D). Furthermore, the population of TGF-βR1/2⁺ TANs decreased following NCAPG knockdown, suggesting NCAPG-mediated TGF-β1 release is associated with TAN recruitment and maintenance (Fig. 2E). Finally, administration of recombinant TGF-β1 (rTGF-β1) to NC^KD^ tumor-bearing mice rescued the malignant phenotype, accelerating tumor growth (Fig. 2F-H) and decreasing cleaved caspase-3 levels (Fig. 2I). This tumor recurrence was accompanied by a repopulation of N2 TANs and a suppression of cytotoxic TNF-α⁺/GZMB⁺ CD8⁺ T cells (Fig. 2J-L).

### NCAPG promotes TGF-β-dependent NETosis to upregulate PD-L1 and suppress CD8⁺ T cell cytotoxicity

NETs extruded by activated neutrophils play crucial roles in the malignant progression and immune suppression in human cancers. We investigated the impact of NCAPG on neutrophil effector functions. Exposure of neutrophils to LUSC CM robustly induced the expression of key NETosis markers, MPO and PAD4, and triggered the release of citrullinated histone H3 (cit-H3)-positive extracellular traps. Importantly, these effects were abrogated when neutrophils were cultured in CM from NCAPG-depleted cells (Fig. S2M-N). Mechanistically, exogenous TGF-β recapitulated the NET-inducing effects of LUSC CM. Conversely, blockade of TGF-β signaling using the receptor inhibitor SB431542 completely nullified this induction, confirming that NCAPG drives NETosis primarily via the TGF-β axis (Fig. S2O-P). Given that NETs can foster an immunosuppressive niche by stabilizing PD-L1 29, we examined this link in our model. *In vivo*, NCAPG depletion significantly reduced intratumoral NETs (Fig. 3A-C). *In vitro*, LUSC CM-induced NETosis correlated with PD-L1 upregulation on tumor cells, a phenotype that was reversed by either NCAPG silencing or TGF-β receptor blockade (Fig. S2Q-R).

To directly assess immune function, we isolated N1 and N2 TAN subsets from mouse tumor tissues for T cell co-culture assays. N2 TANs exhibited a distinct immunosuppressive profile, marked by elevated levels of Arginase-1, NO, and ROS (Fig. 3D). Functionally, while N1 TANs supported CD8⁺ T cell proliferation and activation, N2 TANs potently suppressed T cell expansion and impaired their cytotoxic capacity against KLN205 targets (Fig. 3E-H). To validate the pathogenic role of NETs in vivo, we treated rTGF-β1-supplemented mice with the PAD4 inhibitor GSK484 to block histone citrullination. Pharmacological inhibition of NETosis effectively neutralized the tumor-promoting effects of TGF-β, retarding tumor growth (Fig. 3I-J) and restoring intratumoral CD8⁺ T cell infiltration and effector function (Fig. 3K-O).

### E2F8 transcriptionally activates NCAPG to drive tumorigenesis and dictate neutrophil plasticity

To identify the upstream driver of NCAPG upregulation, we performed an integrated analysis of the UCSC and UALCAN databases 30. Among 11 candidate transcription factors, E2F8 emerged as the top candidate, exhibiting the strongest association with NCAPG expression (Fig. S3A-D). Consistent with this, E2F8 was substantially upregulated in LUSC tissues and cell lines, paralleling the expression pattern of NCAPG (Fig. S3C-G). Furthermore, we induced E2F8 knockdown in the two LUSC cell lines, which led to a reduction in NCAPG expression (Fig. S3H). Luciferase reporter assays with the NCAPG promoter element confirmed that E2F8 reduction impaired the transcriptional activity of NCAPG (Fig. S3I). To further validate the binding relationship between E2F8 and the NCAPG promoter, a ChIP-qPCR assay was conducted, which confirmed that E2F8 binds to the NCAPG promoter (Fig. S3J). We also used the JASPAR database (http://jaspar.genereg.net/) 31 to query potential E2F8 binding sites on the NCAPG promoter. The analysis identified three binding sites with significant binding potential. To validate these predictions, we constructed pGL3-promoter luciferase reporter vectors containing the wild-type (wt) sequence as well as deletion mutants for each binding site: del-#1 (binding site 1 deleted), del-#2 (binding site 2 deleted), and del-#3 (binding site 3 deleted). These reporter constructs were co-transfected with E2F8 into 293T cells. The results revealed that luciferase activity was most significantly reduced in the del-#3 construct, indicating that E2F8 preferentially binds to binding site 3 on the NCAPG promoter (Fig. S3K-L).

Furthermore, NCAPG overexpression (NCAPG^OE^) was introduced into NCI-H226 and NCI-H1703 cells with E2F8 knockdown, which successfully rescued NCAPG expression (Fig. S4A). It was observed that E2F8 knockdown inhibited cell proliferation, an effect reversed by NCAPG upregulation (Fig. S4B-C). Additionally, apoptosis induced by E2F8 knockdown was attenuated by NCAPG overexpression (Fig. S4D). Furthermore, NCAPG overexpression restored migration and invasiveness that were restricted by E2F8 knockdown (Fig. S4E-F). The p-SMAD2 and p-SMAD3 levels in the cells were decreased after E2F8 reduction, but these effects were rescued with NCAPG overexpression (Fig. S4G). Regarding the TAN phenotype, E2F8 knockdown in LUSC cells inhibited N2 polarization, resulting in decreased secretion of IL-8 and VEGFA, alongside an increase in TNF-α secretion (Fig. S4H). In neutrophils, Arg-1 decreased, while iNOS expression increased, with CD95 levels rising and CD184 levels falling when E2F8 was knocked down in LUSC cells (Fig. S4I-M). NET formation was substantially reduced in the E2F8 knockdown condition as well. However, all these changes were reversed when NCAPG was overexpressed in LUSC cells (Fig. S4H-M).

The effects of E2F8 and NCAPG were further confirmed in animal models. KLN205 mouse lung cancer cells were subjected to E2F8^KD^ and NCAPG^OE^ transfections and then implanted into DBA/2 mice. Notably, E2F8 knockdown inhibited the growth of the isogeneic tumors and reduced tumor weight in mice. However, NCAPG overexpression restored tumor growth, effectively reversing the effects of E2F8 knockdown (Fig. 4A-B). Further analysis of TAN polarization in tumor tissues revealed that E2F8 knockdown led to an increase in iNOS^+^Ly6G^+^ (N1) cells and a decrease in Arg1^+^Ly6G^+^ (N2) cell numbers. In contrast, NCAPG overexpression promoted TAN N2 polarization in the tumor microenvironment (Fig. 4C-D). The levels of KC (IL-8) and VEGFA in tumor tissues were also significantly reduced by E2F8 knockdown and rescued following NCAPG overexpression (Fig. 4E). Analysis of TGF-β signaling in tumor tissues indicated that E2F8 reduction led to significantly decreased levels of p-SMAD2 and p-SMAD3, while NCAPG overexpression restored this signaling transduction (Fig. 4F). Furthermore, E2F8 knockdown resulted in an increased number of apoptotic cells, which was alleviated by NCAPG overexpression (Fig. 4G). IHC confirmed that E2F8^KD^ induced lower expression of E2F8 and NCAPG in tumor tissues, while NCAPG^OE^ led to a rise in NCAPG expression (Fig. 4H). Furthermore, E2F8 knockdown in tumor tissues increased the numbers of total CD8^+^ T cells and TNF-α⁺ and GZMB⁺ CD8⁺ T cells, whereas subsequent NCAPG overexpression reversed these effects (Fig. 4I-K). Additionally, to evaluate systemic tumor colonization and overall survival, a separate cohort of DBA/2 mice were administered KLN205 cells with E2F8^KD^ and NCAPG^OE^ transfections via the tail vein. Notably, a marked increase in the 70-day survival rate was observed in mice injected with E2F8^KD^ compared to the control group. However, upon further NCAPG overexpression in these cells, the survival rate of mice was significantly decreased (Fig. 4L).

To further substantiate the role of the E2F8-NCAPG cascade in a setting that closely mimics the spontaneous evolution and natural immune infiltration of human LUSC, a mouse primary LUSC model was established via topical administration of NTCU, followed by administration of specific siRNAs targeting NCAPG or E2F8 after week 30 (Fig. 5A). Notably, inhibition of either NCAPG or E2F8 reduced NTCU-induced nodule formation in the mouse lungs (Fig. 5B-C), accompanied by decreased numbers of Ki67-positive and PD-L1-positive cells in the tumor tissues (Fig. 5D-E). Consistent with the findings above, the population of N2 TANs in tumor tissues was reduced while the number of N1 TANs was increased upon NCAPG or E2F8 inhibition (Fig. 5F-G), and the number of NETs was reduced as well (Fig. 5H). Similarly, in the tail vein injection model, increased numbers of CD8⁺ T cells and TNF-α⁺ and GZMB⁺ CD8⁺ T cells were observed in metastatic nodules formed by KLN205 in lung tissues following E2F8 or NCAPG knockdown (Fig. 5I-K), which also significantly prolonged mouse survival (Fig. 5L).

To empirically confirm the *in vivo* delivery efficiency of the siRNAs, we assessed target expression within the harvested lung nodules. IHC analyses confirmed a robust and significant knockdown of E2F8 and NCAPG in their respective treatment cohorts compared to vehicle controls (Fig. 5M-N). To assess potential non-specific knockdown caused by systemic siRNA delivery, we further examined E2F8 and NCAPG expression in liver tissues. No significant reduction in either E2F8 or NCAPG expression was observed in the liver after tail-vein administration of the corresponding siRNAs, suggesting limited detectable off-target knockdown in this organ under the current experimental conditions (Fig. 5O-P).

### Arundanine targets NCAPG to reduce TGF-β-mediated NET formation and potentiates anti-PD-1 mAb therapy

Prior results highlighted the critical role of NCAPG in LUSC progression; however, there was no FDA-approved or clinically tested drugs targeting NCAPG. Virtual docking using VINA was used to screen 1086 natural compounds potentially targeting NCAPG, from which the top 5 with the lowest binding energies were selected: Arundanine, Songorine, Isolysergic acid, Lysergic acid, and Cinereapyrrole B (Fig. S5A-E). Literature-reported IC50 values for these compounds were applied to NCI-H226 and NCI-H1703 cells. Only Arundanine treatment significantly reduced NCAPG protein levels (Fig. S6A-B). Further analysis revealed that Arundanine markedly decreased NCAPG protein stability (Fig. S6C) and dose-dependently suppressed TGF-β1 production (Fig. S6D-E). To delineate the degradation pathway, cells were co-treated with specific inhibitors. The proteasome inhibitor MG132 completely rescued Arundanine-induced NCAPG downregulation, whereas the lysosome inhibitor Chloroquine (CQ) showed no effect (Fig. S6F). Consistent with proteasomal degradation, immunoprecipitation assays demonstrated that Arundanine treatment substantially increased the polyubiquitination of NCAPG (Fig. S6G).

Treatment of NCI-H226 and NCI-H1703 cells with Arundanine significantly restricted cell proliferation and migration (Fig. S6H-K). To further confirm the safety of Arundanine, mice were administered Arundanine (0, 5, and 10 mg/kg). We observed that the body weight of mice was not affected by Arundanine treatment, and no organ damage was observed (Fig. S6L-R). To biophysically validate the direct interaction predicted by molecular docking, we performed a Cellular Thermal Shift Assay (CETSA) in intact LUSC cells. Arundanine treatment significantly reduced the thermal stability of NCAPG at higher temperatures compared to the vehicle control (Fig. S6S).

Given that direct cytotoxic agents frequently modulate tumor immunogenicity, we investigated whether Arundanine's anti-proliferative effects triggered Immunogenic Cell Death (ICD). Flow cytometry confirmed that Arundanine treatment significantly upregulated the cell-surface exposure of calreticulin (CRT), a classical 'eat-me' signal, on both NCI-H226 and NCI-H1703 cells (Fig. S6T). Furthermore, Arundanine dose-dependently induced the extracellular release of key damage-associated molecular patterns (DAMPs), including High Mobility Group Box 1 (HMGB1) and ATP, into the culture supernatant (Fig. S6U-V). This indicates that Arundanine exerts a dual therapeutic effect: actively reversing NCAPG-mediated suppression while simultaneously inducing ICD to prime anti-tumor immunity.

KLN205 cells were implanted into DBA/2 mice subcutaneously, followed by Arundanine administration (0/5/10 mg/kg) starting on day 8. Arundanine significantly suppressed *in vivo* KLN205 tumor growth (Fig. S7A-B), enhanced CD8^+^ T cell infiltration and cytotoxicity against KLN205 cells (Fig. S7C-F), reduced N2 TAN numbers (Fig. S7G), and inhibited NET formation (Fig. S7H). For the drug-target rescue experiments, NCI-H226 and NCI-H1703 cells stably overexpressing NCAPG (NCAPG^OE^), alongside their respective vector controls, were treated with 10 mM Arundanine. Subsequent cell viability, migration, and TGF-β1 secretion were assessed as described above to evaluate the abrogation of Arundanine-induced effects (Fig. S7I-K).

To evaluate the clinical potential of Arundanine in LUSC treatment, mice intravenously injected with KLN205 cells received Arundanine (10 mg/kg), PD-1 mAb (10 µg/kg), or combination therapy. Both Arundanine and PD-1 mAb significantly restricted tumor nodule formation in mouse lungs (Fig. 6A-B). Arundanine markedly inhibited* in vivo* KLN205 growth and reduced PD-L1 expression (Fig. 6C-D). Consistent with subcutaneous models, Arundanine increased CD8 T cell numbers, including GZMB⁺ and TNF-α⁺ subsets, and enhanced the effect of PD-1 blockade on exhausted T cells (Fig. 6E-H). To further validate the reversal of T cell exhaustion, we evaluated the expression of classical co-inhibitory receptors. Flow cytometry analysis revealed that the combination of Arundanine and PD-1 mAb significantly downregulated the expression of both LAG-3 and TIM-3 on tumor-infiltrating CD8⁺ T cells compared to the vehicle and monotherapy groups (Fig. 6I-J). Arundanine also suppressed N2 TAN infiltration (Fig. 6K), reduced NET formation (Fig. 6L), and lowered TGF-β1 levels (Fig. 6M). Combination therapy with Arundanine and PD-1 mAb significantly prolonged survival in KLN205-inoculated mice (Fig. 6N). These findings indicated that Arundanine holds potential to sensitize LUSC to PD-1 mAb therapy in clinical settings.

### E2F8 and NCAPG may serve as potential targets for LUSC treatment

To further analyze the clinical relevance of E2F8 and NCAPG in LUSC, we acquired TMAs containing 87 pairs of LUSC and adjacent tissue samples. Notably, both E2F8 and NCAPG showed higher H-scores in the tumor tissue samples versus the adjacent tissue samples (Fig. 7A-C). Based on the median H-scores, the samples were allocated into high- or low-E2F8 and NCAPG groups. Importantly, the increased H-scores of either E2F8 or NCAPG were found to be linked to increased staining of PD-L1 and MPO (Fig. 7D-G). Among the 87 sample donors, 17 underwent anti-PD-1 therapy, including 7 non-responders and 10 responders. Notably, the H-scores of E2F8 and NCAPG were higher in non-responders than in responders (Fig. 7H-I). Given the limited sample size of this clinical subgroup, these specific observations remain exploratory and suggest a potential, rather than definitive, association between E2F8/NCAPG expression and PD-1 blockade resistance.

## Discussion

The current treatment options for LUSC face significant challenges due to the inherent tumor heterogeneity, lack of well-defined oncogenic mutations, limited insights into the mechanisms of oncogenic pathways, and a deficiency in effective animal models 32, 33. In this study, we report an E2F8/NCAPG cascade that is upregulated in LUSC, which appears to exert a crucial role in enhancing the malignant characteristics of LUSC tumor cells, particularly through the TGF-β pathway and the polarization of TANs towards a pro-tumor N2 phenotype. Arundanine, an alkaloid targeting NCAPG and modulating its degradation, may offer a promising therapeutic strategy.

Differential gene expression analysis using publicly accessible datasets has proven beneficial for pinpointing potential therapeutic targets 34, 35. This study employed bioinformatics techniques using the GEO GSE31552 dataset and the GEPIA platform, leading to the identification of NCAPG as a gene with abnormal expression levels in LUSC tissues. Specifically, we observed elevated transcription levels of NCAPG in NCI-H226 and NCI-H1703 when compared to normal BEAS-2B cells. The increasing recognition of NCAPG as a critical player across various cancers is a key finding in recent cancer research. Elevated NCAPG expression has been associated with unfavorable prognosis and enhanced tumor progression in multiple cancer types, including lung cancer 36-39. Sun and colleagues' work, which identified NCAPG upregulation as an independent risk factor for NSCLC, partly aligns with our study 11. Their study demonstrated that downregulation of NCAPG inhibited tumor expansion and dissemination in both cellular and animal models. Additionally, NCAPG's association with lung cancer progression was noted in LUAD, where elevated expression has been linked to dismal patient outcomes 40, 41. Our results support the critical function of NCAPG in LUSC advancement, as silencing NCAPG expression in LUSC cells significantly reduced their proliferation and metastatic spread. Importantly, NCAPG may also influence immune cell infiltration in NSCLC 42, as it activates the immunosuppressive TGF-β pathway, facilitating LUAD advancement 13. This dynamic is also relevant in LUSC. Our data suggested that NCAPG knockdown in LUSC cells restricted the phosphorylation of key TGF-β signaling proteins, SMAD2 and SMAD3. The presence of TGF-β within the tumor microenvironment is known to promote neutrophil polarization towards a more pro-tumor phenotype 43. Bioinformatics analyses from our study demonstrated an association between NCAPG levels and neutrophil infiltration in LUSC. Functional assays further demonstrated that CM from LUSC cells lacking NCAPG resulted in an N2-to-N1 shift in neutrophils* in vitro*, along with decreased concentrations of immunosuppressive cytokines. NETs formed following neutrophil death have been reported to participate in many processes during cancer progression 44, 45. NET formation has also been found to awaken dormant cancer cells 17. Studies have also indicated that NETs can contribute to an immunosuppressive environment by promoting PD-L1 signaling 44-46. Here, we identified that stimulation with LUSC CM significantly increased the expression of NET formation markers in neutrophils, with the effects negated by TGF-β receptor blockade. Additionally, NCAPG knockdown reduced tumorigenic activity of KLN205 cells, reduced the staining for PD-L1 while enhancing staining for cleaved caspase-3, accompanied by increased population of effector (TNF-α⁺ and GZMB⁺) CD8^+^ T cells. These findings confirm that NCAPG not only promotes LUSC progression but also contributes to immune suppression through TGF-β pathway activation.

To identify the upstream regulator driving the abnormal transcription of NCAPG in LUSC, E2F8 was identified as a key candidate through comprehensive bioinformatics analysis. E2F8 belongs to the E2F family, which constitutes essential components of the transcriptional machinery crucial for embryogenesis 47. Recent studies have highlighted its significant roles in regulating cell cycle dynamics, differentiation, proliferation, and apoptosis upon varied environmental signals 48. For instance, research by Park *et al.* in 2015 established that irregular upregulation of E2F8 in LUAD correlated with poor prognoses for patients 49. Subsequently, Liu* et al.* reported in 2023 that E2F8 facilitated proliferation while inhibiting apoptosis in LUAD cells by triggering the expression of ribonucleotide reductase regulatory subunit M2 50. Another member of the E2F family, E2F7, has shown associations with infiltrating immune cells, notably neutrophils, in LUAD 51. However, the roles of E2F8 in LUSC progression, particularly concerning neutrophil phenotypic changes, have yet to be elucidated. Here, we report that E2F8 binds to the NCAPG promoter, activating its transcription. Functional assays further revealed that depleting E2F8 diminished NCAPG transcription, leading to reduced aggressiveness of LUSC cells. Additionally, the CM from LUSC cells with E2F8 knockdown showed a decrease in N2 skewing of TANs. These effects were reversed upon restoring NCAPG expression. Additionally, the* in vivo* experiments indicated that NCAPG and E2F8 supported tumorigenesis and N2 polarization in both isogeneic tumors and NTCU-induced primary LUSC models, with reduced tumorigenesis, NETosis, and CD8^+^ T cell exhaustion identified in tumors formed upon NCAPG or E2F8 knockdown. This evidence further underscores the correlation between E2F8-driven NCAPG transcription and progression of LUSC, as well as neutrophil polarization via TGF-β signaling. Furthermore, by analyzing LUSC TMA, we identified increased E2F8 and NCAPG expression in clinical LUSC samples, with their upregulation linked to increased NET formation and immunosuppression.

Molecular docking was carried out to identify potential compounds targeting NCAPG, with Arundanine as a candidate. Arundanine is a dimeric indole alkaloid isolated from the roots of *Arundo donax* L. (giant reed, Poaceae family). It is structurally related to other bis-indole alkaloids in the plant, featuring a 4-(indol-1-yl)-5-hydroxyindole core system. While the biological function of Arundanine remains elusive, previous evidence has indicated the antiproliferative/antitumor effect of the *Arundo donax* component 52. Importantly, we verified that Arundanine directly targeted NCAPG (CETSA) and reduced its protein stability through ubiquitination, which subsequently suppressed TGF-β activity, NET formation, N2 TAN skewing, and improved CD8^+^ T cell cytotoxicity in both cell and animal experiments, suggesting a potential tumor-suppressive and immunomodulatory function of Arundanine in LUSC. Indeed, in the tail vein injection model, Arundanine potentiated the anti-tumor functions of the PD-1 mAb, reducing tumor metastasis, enhancing immune activity, and extending animal survival. In vitro, Arundanine treatment directly suppressed NCI-H226 and NCI-H1703 cell proliferation, increased surface exposure of CRT, and elevated the release of HMGB1 and ATP.

Several limitations should be acknowledged. First, while our findings establish Arundanine as a modulator of the E2F8/NCAPG axis that successfully reverses immunosuppression, certain translational limitations remain. Specifically, high-resolution pharmacokinetic profiles characterizing plasma clearance and intratumoral drug accumulation, alongside broad off-target screening panels, were not conducted here. Nevertheless, the preliminary in vivo safety profile, evidenced by unchanged body weights, intact tissue histology, and stable serum biomarkers for liver, heart, and kidney function, indicates that the current dosing regimen falls within a safe therapeutic window. Fully elucidating the precise systemic pharmacokinetics of Arundanine remains an active objective of our ongoing translational pipeline. While the LUSC TMA analysis suggested that E2F8 and NCAPG expression in clinical LUSC samples was associated with increased NET formation and immunosuppression, the clinical TMA cohort was relatively small, particularly the anti-PD-1-treated subgroup of 17 patients. Thus, the observed associations with PD-1 blockade response should be considered exploratory. Larger prospective cohorts are needed to validate the clinical and predictive value of E2F8 and NCAPG in LUSC.

In summary, evidence derived from bioinformatics data, cellular and animal studies, and TMA analyses, underscores the significance of the E2F8-NCAPG interaction in the development and advancement of LUSC via TGF-β pathway activation and N2 TAN skewing. Targeting NCAPG with Arundanine reduces LUSC progression and sensitizes LUSC to PD-1 blockade, offering a promising therapeutic strategy for LUSC management.

## Supplementary Material

Supplementary figures and table.

## Figures and Tables

**Figure 1:**
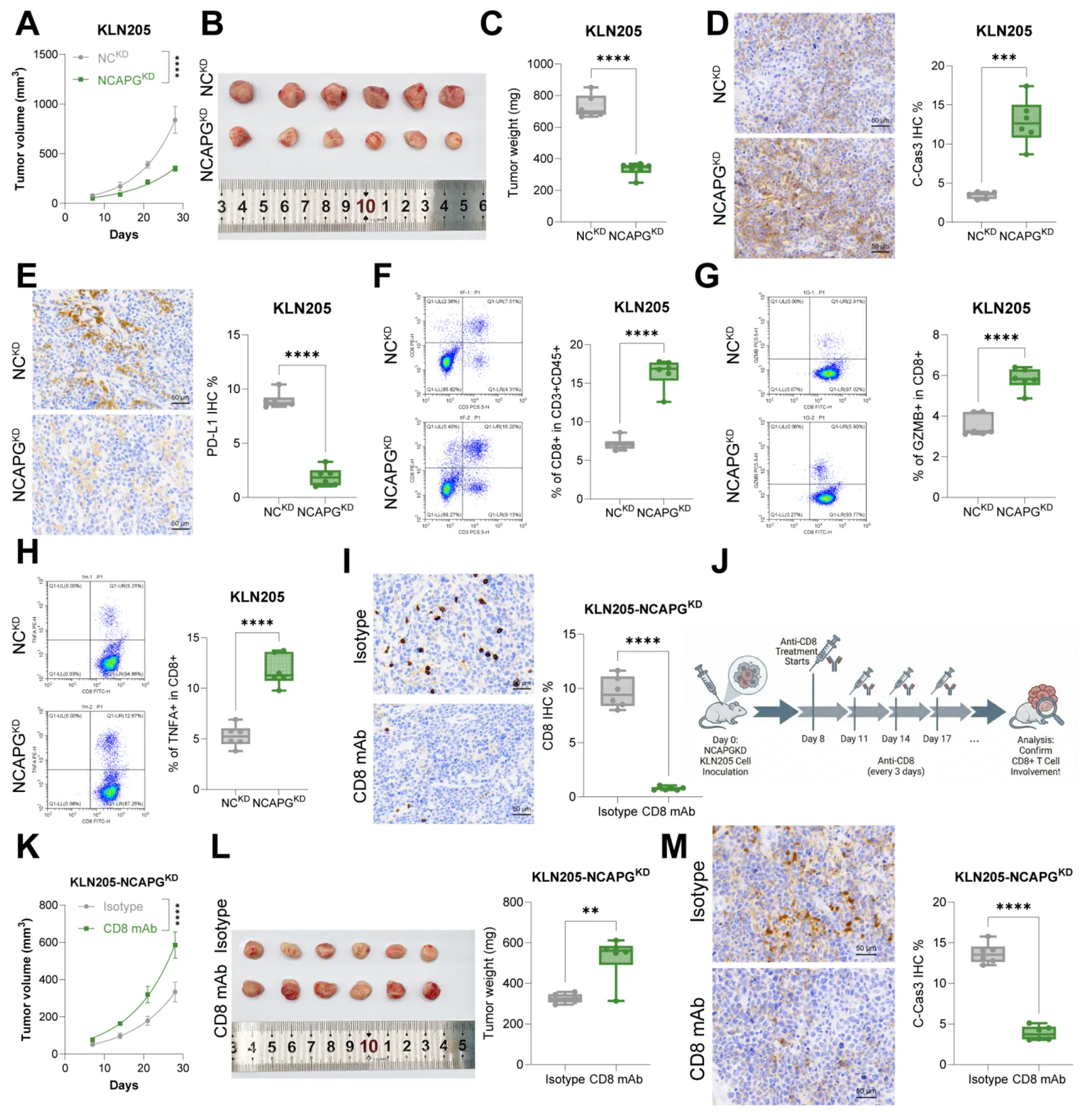
NCAPG knockdown reduces tumorigenic activity of KLN205 cells *in vivo* and upregulates effector CD8^+^ T cell functional markers. KLN205 cells with stable NCAPG knockdown were implanted into DBA/2 mice subcutaneously. (A) Growth rate of subcutaneous tumors in mice over 28 days. (B-C) Representative images and weights of subcutaneous tumors in mice on day 28. (D-E) IHC detection of staining intensity for cleaved caspase-3 and PD-L1 in tumor tissues. (F-H) Flow cytometry detection of the number of CD3⁺CD45⁺CD8⁺ (F) cells, as well as CD8⁺ GZMB⁺ (G) and CD8⁺ TNF-α⁺ (H) T cells in tumor tissues. (I) IHC detection of CD8⁺ cells in tumor tissues. (J) Anti-CD8 treatment was administered every 3 days starting on day 8 after inoculation of NCAPG^KD^ KLN205 cells. (K) Growth rate of subcutaneous tumors in mice over 28 days. (L) Representative images and weight of tumors in mice on day 28. (M) IHC detection of cleaved caspase-3 in tumor tissues. Data are expressed as the mean ± SD and shown as dot plots with whiskers representing SD. For animal experiments, n = 6 biologically independent mice were analyzed per group. Data are presented as dot-and-whisker plots. **p < 0.01, ***p < 0.001, ****p < 0.0001.

**Figure 2:**
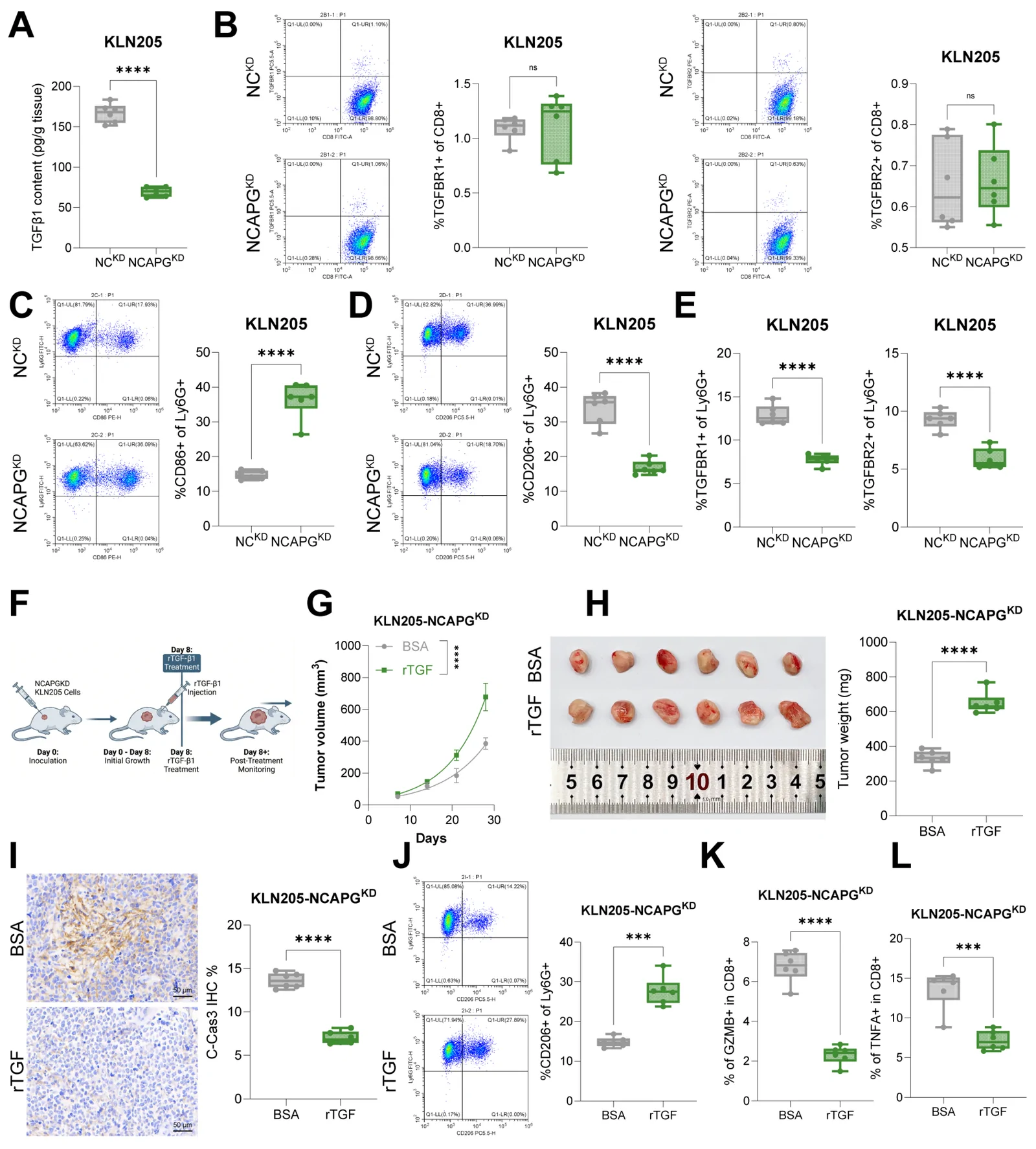
NCAPG knockdown reduces TGF-β1 levels and restricts N2 TAN polarization in subcutaneous tumors. KLN205 cells with stable NCAPG knockdown were implanted into DBA/2 mice subcutaneously. (A) ELISA detection of TGF-β1 levels in mouse tumor tissues. (B) Flow cytometry detection of the number of TGF-βR1⁺ and TGF-βR2⁺ CD8⁺ T cells in tumor tissues. (C-D) Flow cytometry detection of the number of Ly6G⁺CD86⁺ or Ly6G⁺CD206⁺ cells in mouse tumor tissues. (E) Flow cytometry detection of the number of TGF-βR1⁺ and TGF-βR2⁺ Ly6G⁺ TANs in tumor tissues. (F-L) Recombinant TGF-β1 (rTGF-β1) was injected on day 8 in mice inoculated with NCAPG^KD^ KLN205 cells. (G) Growth rate of subcutaneous tumors in mice over 28 days. (H) Representative images and weight of tumors in mice on day 28. (I) IHC detection of cleaved caspase-3 in tumor tissues. (J) Flow cytometry detection of the number of Ly6G⁺CD206⁺ cells in mouse tumor tissues. (K-L) Flow cytometry detection of the number of CD8⁺GZMB⁺ (K) and CD8⁺TNF-α⁺ (L) T cells in tumor tissues. Data are expressed as the mean ± SD and shown as dot plots with whiskers representing SD. In vitro assays represent n = 6 independent biological replicates. For animal experiments, n = 6 biologically independent mice were analyzed per group. ***p < 0.001, ****p < 0.0001.

**Figure 3:**
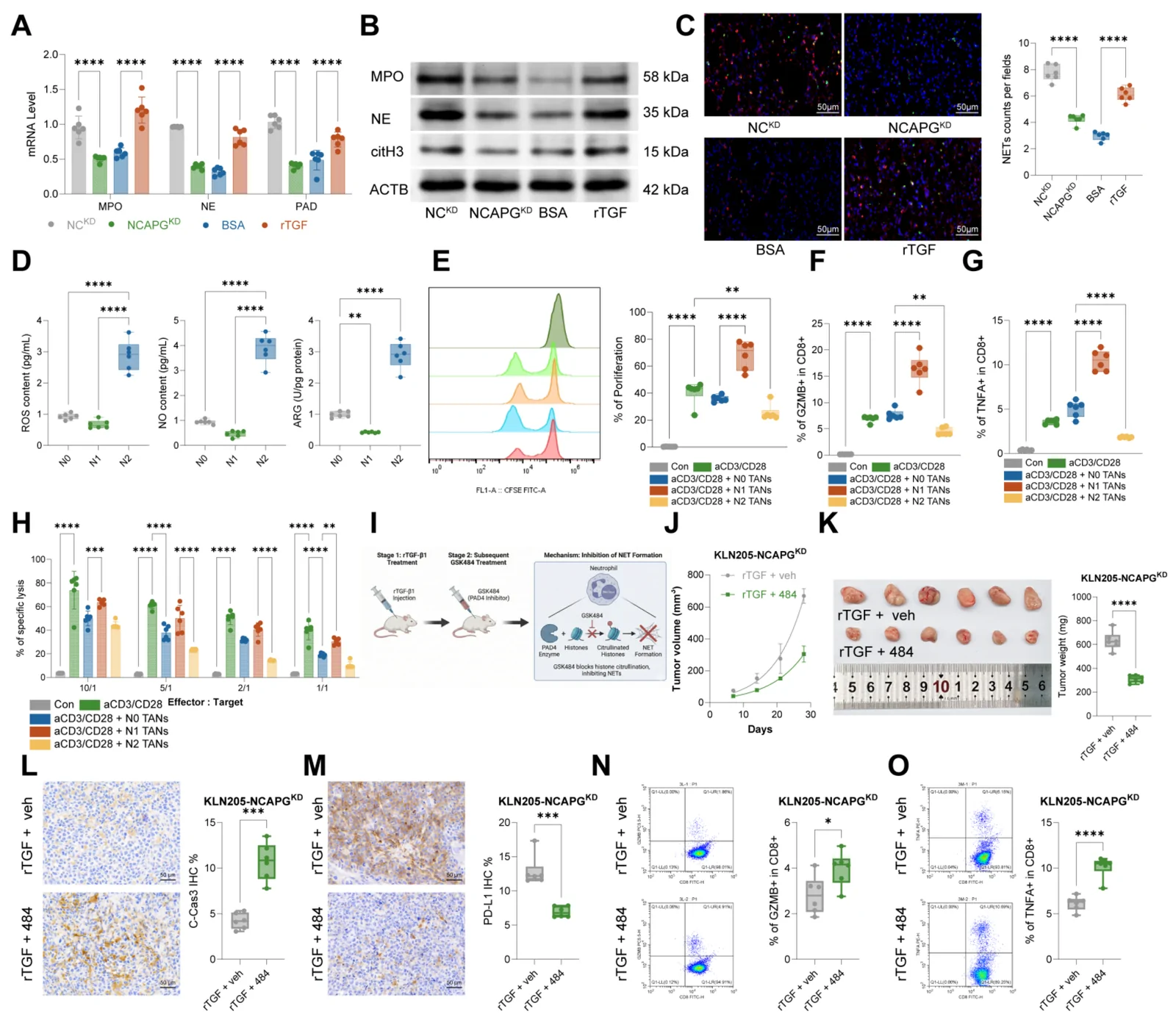
NCAPG promotes NET formation and suppresses CD8^+^ T cell activity. “(A) qPCR analysis of mRNA expression of MPO, NE, and PAD4 in subcutaneous tumors formed by KLN205 cells across groups. (B) WB detection of protein levels of MPO, NE, and cit-H3 in subcutaneous tumors formed by KLN205 cells across groups (C) NETs in tumor tissues determined using immunofluorescence staining of MPO and cit-H3. N0 neutrophils, N1 TANs, and N2 TANs were isolated from subcutaneous tumor tissues. (D) ELISA detection of NO, ROS, and ARG activity in neutrophils. (E-G) N0 Neutrophils, N1 TANs, and N2 TANs were co-cultured with anti-CD3/CD28-stimulated CD8⁺ T cells. (E) Proliferation of CFSE-labeled CD8⁺ T cells post-co-culture determined using flow cytometry. (F-G) Flow cytometry detection of the proportion of GZMB⁺ (F) or TNF-α⁺ (G) CD8⁺ T cells after co-culture. (H) Co-cultured T cells were incubated with KLN205 cells at ratios of 5:1, 2:1, or 1:1, and the cytotoxic effect of CD8⁺ T cells on KLN205 cells was assessed using a lactate dehydrogenase (LDH) assay kit. (I) Mice receiving rTGF-β1 treatment were additionally administered the PAD4 inhibitor GSK484 (to block histone citrullination). (J) Growth rate of subcutaneous tumors in mice over 28 days. (K) Representative images and weight of tumors in mice on day 28. (L-M) IHC detection of staining intensity for cleaved caspase-3 (L) and PD-L1 (M) in tumor tissues. (N-O) Flow cytometry detection of the proportion of GZMB⁺ (N) or TNF-α⁺ (O) CD8⁺ T cells in tumor tissues. Data are expressed as the mean ± SD and shown as dot plots with whiskers representing SD. In vitro assays represent n = 6 independent biological replicates. For animal experiments, n = 6 biologically independent mice were analyzed per group. *p < 0.05, **p < 0.01, ***p < 0.001, ****p < 0.0001.

**Figure 4:**
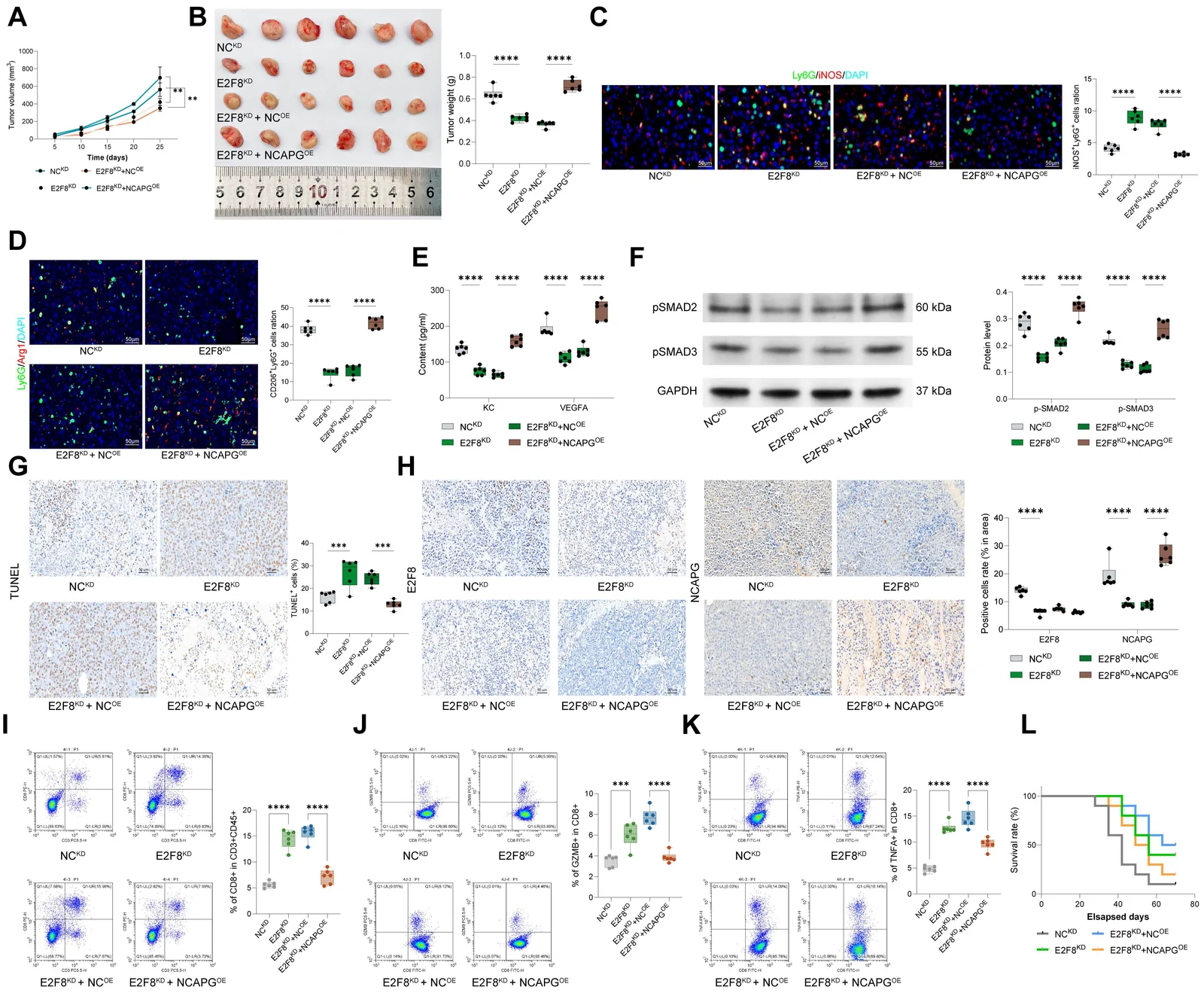
The E2F8/NCAPG axis affects TAN phenotype and tumorigenesis in mice. KLN205 cells stably transfected with E2F8^KD^ and NCAPG^OE^ were implanted into DBA/2 mice subcutaneously. (A) Growth rate of subcutaneous tumors in mice over 28 days. (B) Representative images and weight of in mice on day 28. (C-D) Populations of iNOS^+^Ly6G^+^ (N1) and Arg1^+^Ly6G^+^ (N2) neutrophils in tumor tissues determined using double label immunofluorescence staining. (E) Concentrations of IL-8 and VEGFA in tumors determined using ELISA kits. (F) Levels of p-SMAD2 and p-SMAD3 in tumors determined using WB analysis. (G) Cell apoptosis in tumors determined using TUNEL assay. (H) Expression of E2F8 and NCAPG in tumor tissues analyzed using IHC. (I-K) Flow cytometry detection of the proportion of total CD3⁺CD45⁺CD8⁺ T cells (I), GZMB⁺ (J) or TNF-α⁺ CD8⁺ (K) T cells in tumor tissues. (L) KLN205 mouse lung cancer cells were subjected to E2F8^KD^ and NCAPG^OE^ transfections and then implanted into DBA/2 mice via tail vein injection, followed by Kaplan-Meier analysis for 70-day survival rate of mice in each group. Data are expressed as the mean ± SD and shown as dot plots with whiskers representing SD. In vitro assays represent n = 6 independent biological replicates. For animal experiments, n = 6 biologically independent mice were analyzed per group, except n = 10 per group in panel L. **p < 0.01, ***p < 0.001, ****p < 0.0001.

**Figure 5:**
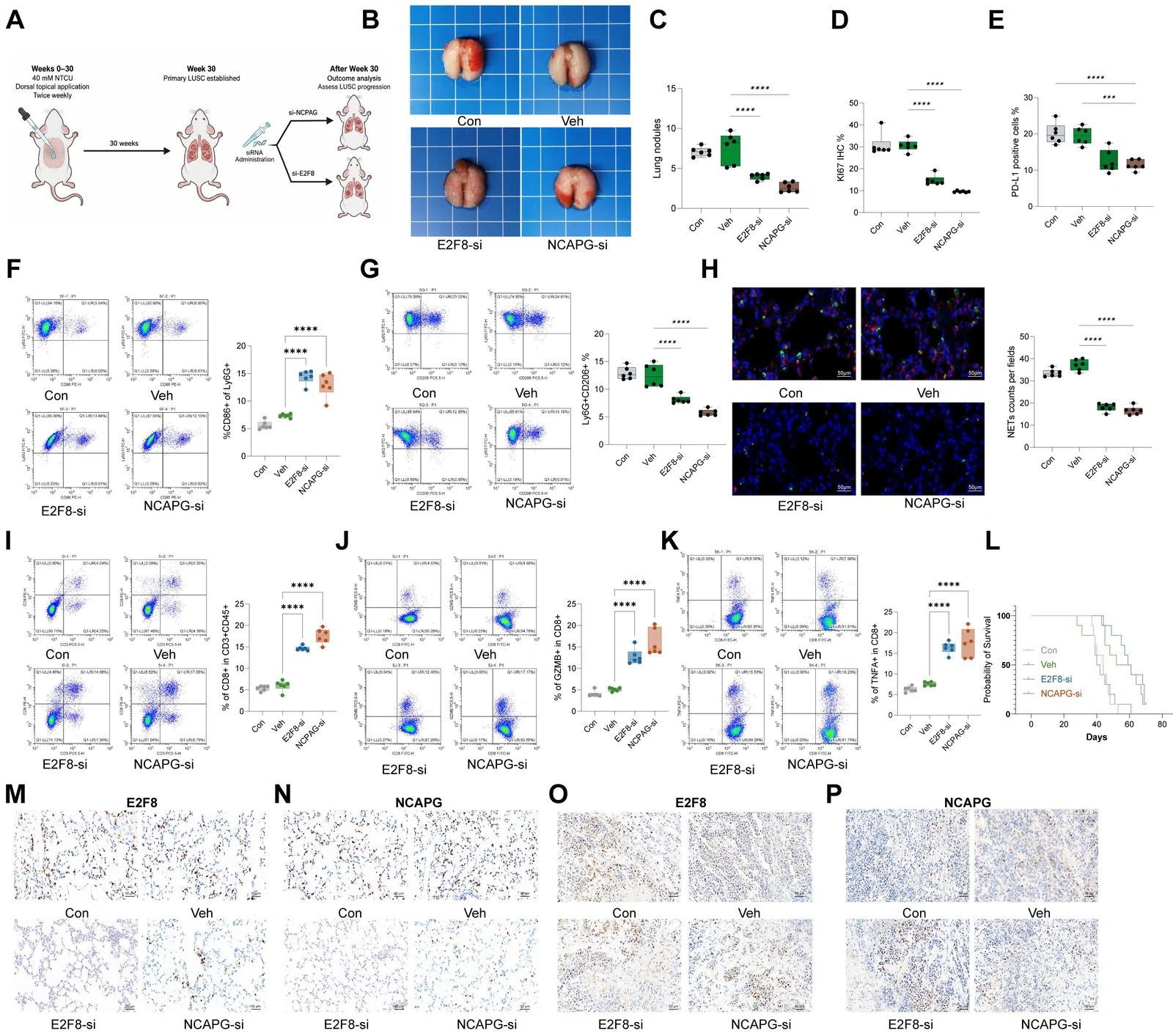
Blocking E2F8 or NCAPG reduces NTCU-induced LUSC development and metastasis in mice. (A) Diagrammatic presentation of the primary LUSC model in BALB/c mice: NTCU (40 mM) was dissolved in acetone and topically applied to the shaved dorsal skin of the mice twice weekly for 30 weeks. Starting from week 30, mice were administered vehicle or specific siRNAs targeting NCAPG or E2F8. (B) Gross images of lung tissues with tumor nodules in each group. (C) Number of tumor nodules in the lung. Positive staining of Ki67 (D) and PD-L1 (E) in tumor tissues determined using IHC. (F-G) Population of N1 TANs (Ly6G^+^CD86^+^) and N2 TANs (Ly6G^+^CD206^+^) in tumors determined using flow cytometry. (H) NETs in tumor tissues determined using immunofluorescence staining of MPO and cit-H3. KLN205 cells stably transfected with siRNAs targeting E2F8 or NCAPG were implanted into DBA/2 mice through the tail vein. (I-K) Flow cytometry detection of the proportion of total CD3⁺CD45⁺CD8⁺ T cells (I), GZMB⁺ (J) or TNF-α⁺ CD8⁺ (K) T cells in metastatic nodules in the lung tissues. (L) Kaplan-Meier analysis for 70-day survival rate of mice in each group. Each group contained six mice. (M-N) Representative IHC images and quantification confirming the in vivo knockdown efficiency of E2F8 (M) and NCAPG (N) within the harvested NTCU-induced lung tumor nodules across the indicated systemic siRNA treatment cohorts compared to vehicle controls. (O-P) Evaluation of E2F8 and NCAPG expression in off-target liver tissues from the treated cohorts to assess the organ specificity and systemic safety of the in vivo siRNA delivery. Data are expressed as the mean ± SD and shown as dot plots with whiskers representing SD. In vitro assays represent n = 6 independent biological replicates. For animal experiments, n = 6 biologically independent mice were analyzed per group, except n = 10 per group in panel L. ***p < 0.001, ****p < 0.0001.

**Figure 6:**
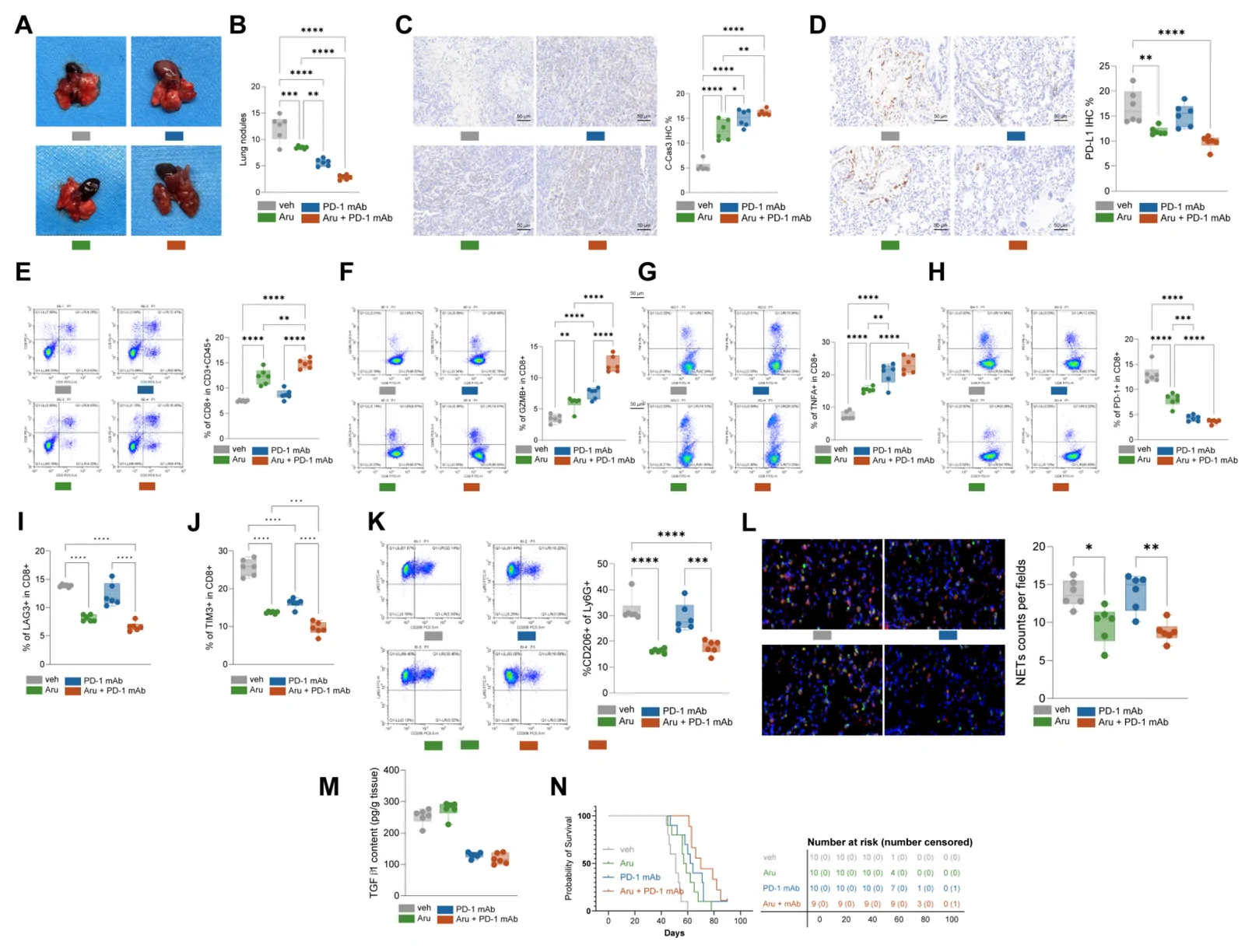
Arundanine targets NCAPG to reduce TGF-β-mediated NET formation and potentiates anti-PD-1 mAb therapy. KLN205 cells were injected into DBA/2 mice via tail vein. Starting from day 8, mice were treated with Arundanine, PD-1 mAb, or Arundanine combined with PD-1 mAb every 3 days until day 36. (A) Gross images of lung tissues with metastatic tumor nodules in each group. (B) Number of tumor nodules in the lung. Positive staining of C-Cas-3 (C) and PD-L1 (D) in tumor tissues determined using IHC. (E-H) Flow cytometry detection of the number of total CD3⁺CD45⁺CD8⁺ T (E), CD8⁺TNF-α⁺CD8⁺ T (F), CD8⁺GZMB⁺CD8⁺ T (G), and PD-1⁺CD8⁺ T (H) cells in tumor tissues. (I-J) Flow cytometric evaluation of classical co-inhibitory receptors on tumor-infiltrating CD8⁺ T cells, demonstrating the quantification of LAG-3 (I) and TIM-3 (J) expression across the vehicle, monotherapy, and Arundanine + PD-1 mAb combination treatment groups. (K) Flow cytometry detection of the number of Ly6G⁺CD206⁺ cells in tumor tissues. (L) Number of NETs in tumor tissues determined using immunofluorescence staining of MPO and cit-H3. (M) ELISA for the content of TGF-β1 in the tumor tissue. (N) Kaplan-Meier analysis of 90-day survival rate for each group. Data are expressed as the mean ± SD and shown as dot plots with whiskers representing SD. In vitro assays represent n = 6 independent biological replicates. For animal experiments, n = 6 biologically independent mice were analyzed per group, except panel N. In panel N, n = 10 in Vehicle, Arundanine, and PD-1 mAb groups and n = 9 in Arundanine + PD-1 mAb group. ***p < 0.001, ****p < 0.0001.

**Figure 7:**
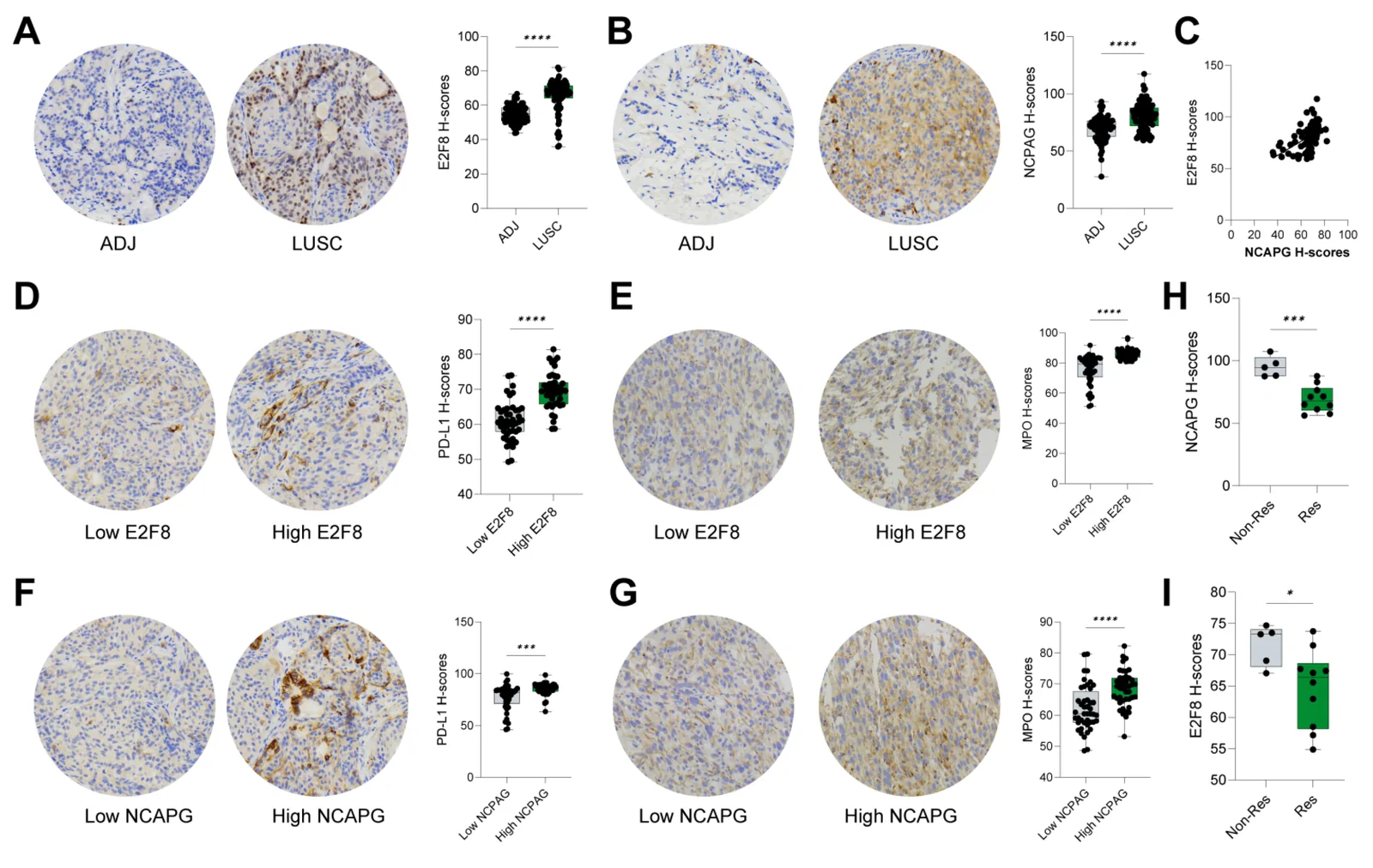
E2F8 and NCAPG serve as potential targets for LUSC treatment. TMAs containing 87 pairs of LUSC and adjacent tissue samples were acquired for analyses. H-scores of E2F8 (A) and NCAPG (B) in LUSC and adjacent tissue samples determined using IHC assays. A positive correlation between E2F8 and NCAPG H-scores in LUSC tissues. The samples were allocated into high- or low-E2F8 and NCAPG groups based on the median H-scores. H-scores of PD-L1 (D) and MPO (E) in high- or low-E2F8 H-score samples determined using IHC. PD-L1 (F) and MPO (G) in high- or low-NCAPG H-score samples determined using IHC. Among the 87 sample donors, 17 underwent anti-PD-1 therapy and were allocated into non-responders (n = 7) and responders (n = 10). H scores of NCAPG (H) and E2F8 (I) in non-responders and responders determined using IHC assays. Data are expressed as the mean ± SD and shown as dot plots with whiskers representing SD. *p < 0.05, ***p < 0.001, ****p < 0.0001.

## Data Availability

Research data supporting this publication are available upon request.
