# Supplementary material for: Targeting NCAPG with Arundanine Suppresses Neutrophil/NET-Mediated Immune Evasion and Enhances CD8+ T Cell Function in Lung Squamous Cell Carcinoma
Source: Int J Biol Sci. 2026 Sep 11;22(15):8211–29. doi: 10.7150/ijbs.134668 (PMC13685603; doi:10.7150/ijbs.134668)
Supplement: Supplementary file 1 [file ijbsv22p8211s1.pdf]

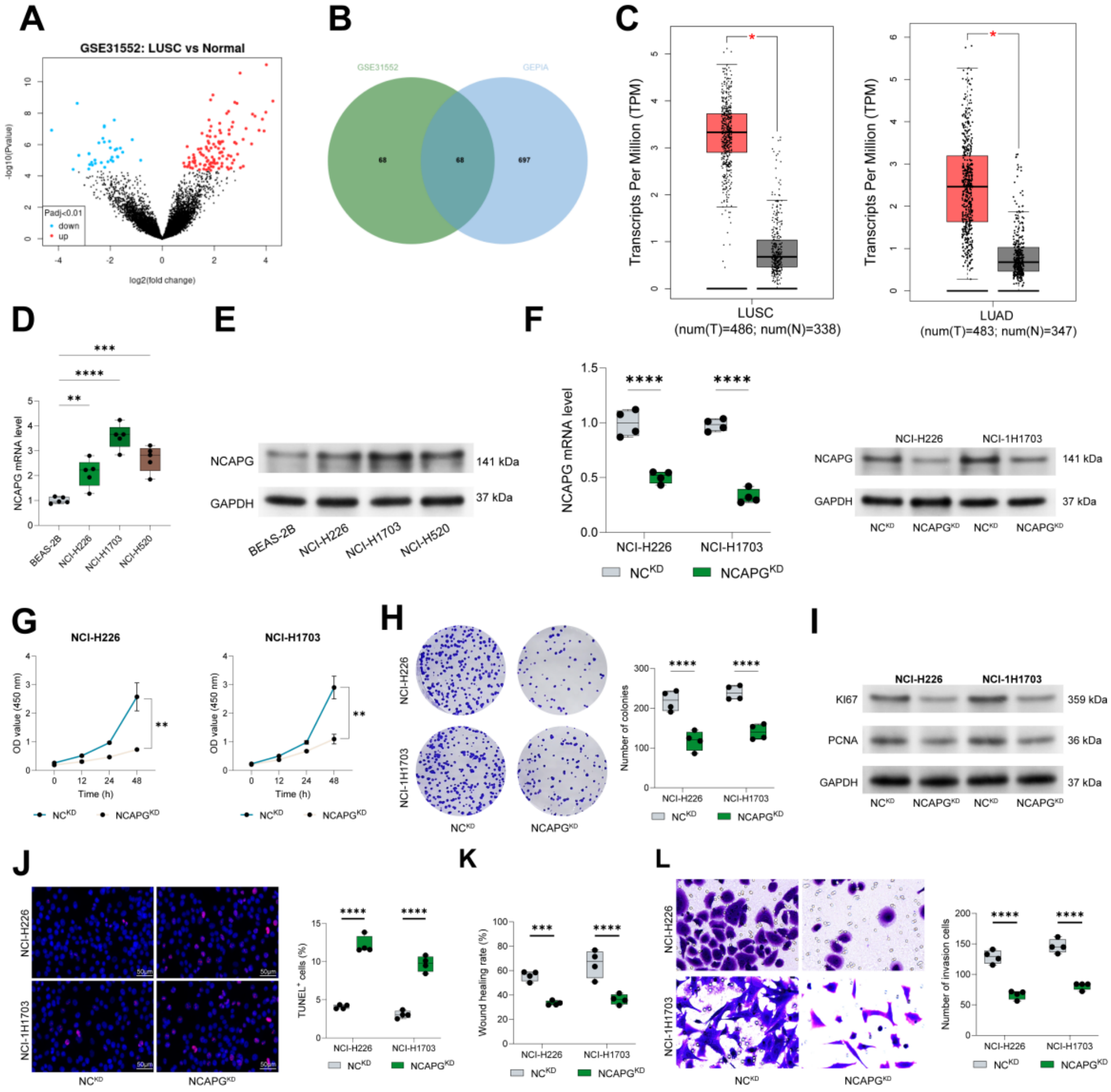

Fig. S1 NCAPG shows aberrant heightened expression in LUSC. (A) Volcano plots for DEGs between LUSC and normal samples in the GSE31552 dataset. (B) Intersections of the DEGs obtained from the GSE31552 dataset and those from the GEPIA system. (C) Expression pattern of NCAPG in LUSC and normal samples in the GEPIA system. mRNA (D) and protein (E) levels of NCAPG in LUSC cell lines (NCI-H226 and NCI-H1703) and normal BEAS-2B cells measured using RT-qPCR and WB analysis. (F) mRNA and protein levels of NCAPG in NCI-H226 and NCI-H1703 cells after transfection of $NCAPG^{KD}$ or NC^{KD} determined using RT-qPCR and WB analysis. (G) Proliferation of cells after transfection analyzed by CCK-8 assays. (H) Colony formation ability of cells after transfection. (I) Protein levels of Ki67 and PCNA in transfected cells analyzed using WB analysis. (J) Apoptosis in transfected cells measured using TUNEL assay. (K) Migration of cells assessed using wound healing assay. (L) Invasion of cells assessed using Transwell assay. Cell experiments were repeated six times. Data are presented as dot-and-whisker plots. $^{**}p < 0.01$, $^{***}p < 0.001$, $^{****}p <$

0.0001. Data are expressed as the mean ± SD and shown as dot plots with whiskers representing SD. In vitro assays represent n = 6 independent biological replicates. For animal experiments, n = 6 biologically independent mice were analyzed per group. ** $p < 0.01$, *** $p < 0.001$, **** $p < 0.0001$.

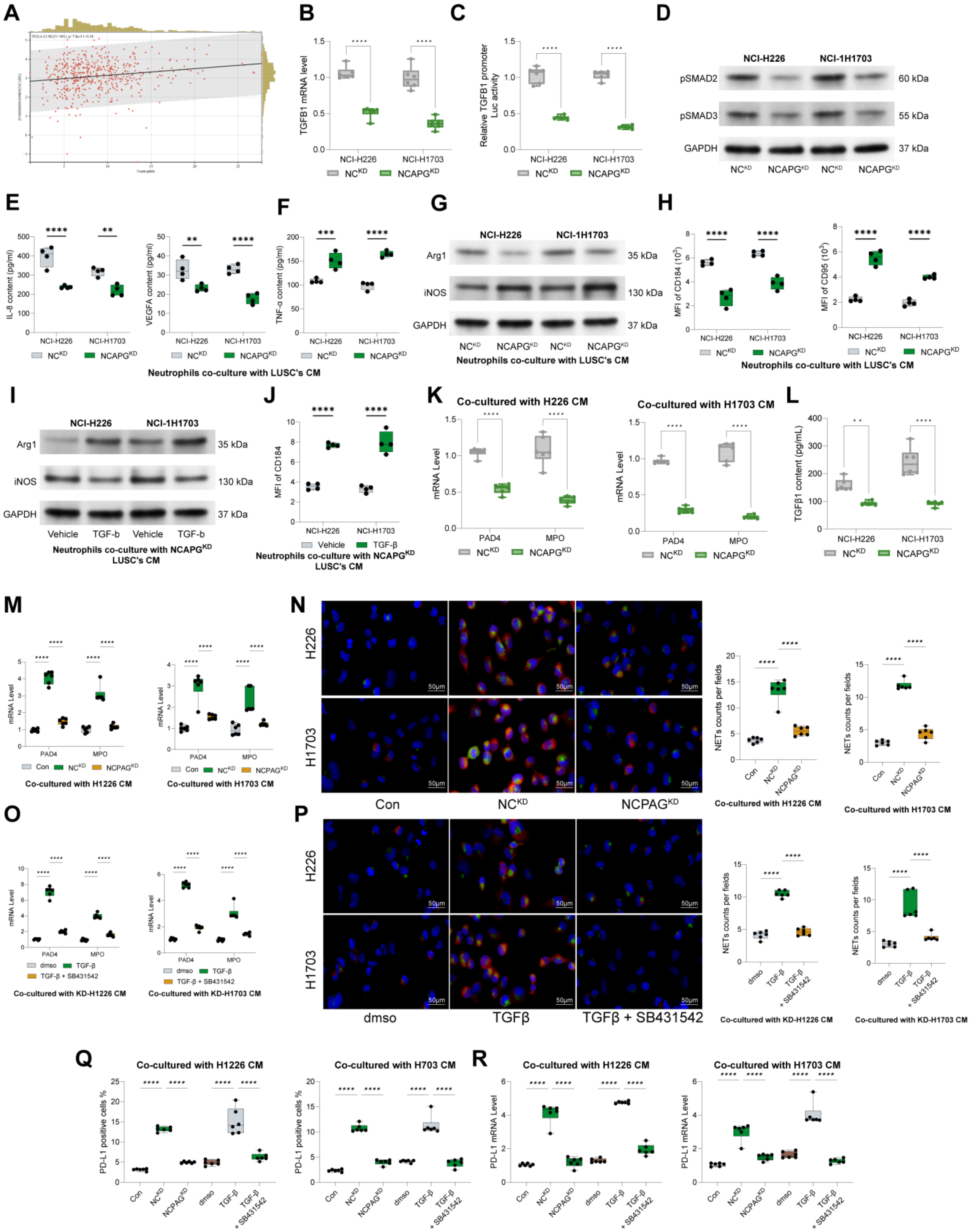

Fig. S2 NCAPG enhances N2 skewing of neutrophils in the context of LUSC through the TGF-β pathway. (A) Correlation between NCAPG and neutrophil infiltration in LUSC predicted using the Sangerbox tool. (B) mRNA expression of TGFb1 in NCI-H226 and NCI-H1703 cells determined using RT-qPCR. (C) Luciferase activity of TGFB1 promoter in $NCAPG^{KD}$ NCI-H226 and NCI-H1703 cells. (D) Levels of p-SMAD2 and p-SMAD3 in LUSC cells with NCAPG knockdown determined using WB analysis. Neutrophils were cultured in CM derived from LUSC cells transfected with $NCAPG^{KD}$ or NC^{KD}. (E-F) Concentrations of IL-8, VEGFA, and TNF-α in the cell culture supernatant determined using ELISA kits. (G) Protein levels of Arg-1 and iNOS in neutrophils after culture in the CM determined using WB analysis. (H) Expression of TAN N1 marker CD95 and N2 marker CD184 in neutrophils after culture in the CM analyzed using flow cytometry. (I) Protein levels of Arg-1 and iNOS in neutrophils after the addition of TGF-β to the culture system determined using WB analysis. (J) Expression of TAN N1 marker CD95 and N2 marker CD184 in neutrophils after the addition of TGF-β to the culture system analyzed using flow cytometry. (K) mRNA expression of PAD4 and MPO in freshly isolated primary human peripheral blood neutrophils following co-culture with CM derived from control or NCAPG-depleted LUSC cells. (L) ELISA quantification of TGF-β1 concentrations in the cell-free culture supernatant of isolated NCI-H226 and NCI-H1703 cells following NCAPG knockdown. (M) mRNA expression of PAD4 and MPO in neutrophils after culture in normal culture system (control) or in CM derived from LUSC cells transfected with $NCAPG^{KD}$ or NC^{KD}, determined using RT-qPCR. (N) NET formation determined using immunofluorescence staining of MPO and cit-H3. The CM derived from $NCAPG^{KD}$ cells were added with TGF-β or the additional TGFBR inhibitor SB431542. (O) mRNA expression of PAD4 and MPO in neutrophils after culture in different conditions determined using RT-qPCR. (P) NET formation determined using immunofluorescence staining of MPO and cit-H3. (Q) PD-L1-positive cells after culture in different conditions determined using flow cytometry. (R) mRNA expression of PD-L1 in cells determined using RT-qPCR. Data are expressed as the mean ± SD and shown as dot plots with whiskers representing SD. In vitro assays represent n = 6 independent biological replicates.

For animal experiments, n = 6 biologically independent mice were analyzed per group. $^{**}p < 0.01$, $^{***}p < 0.001$, $^{****}p < 0.0001$.

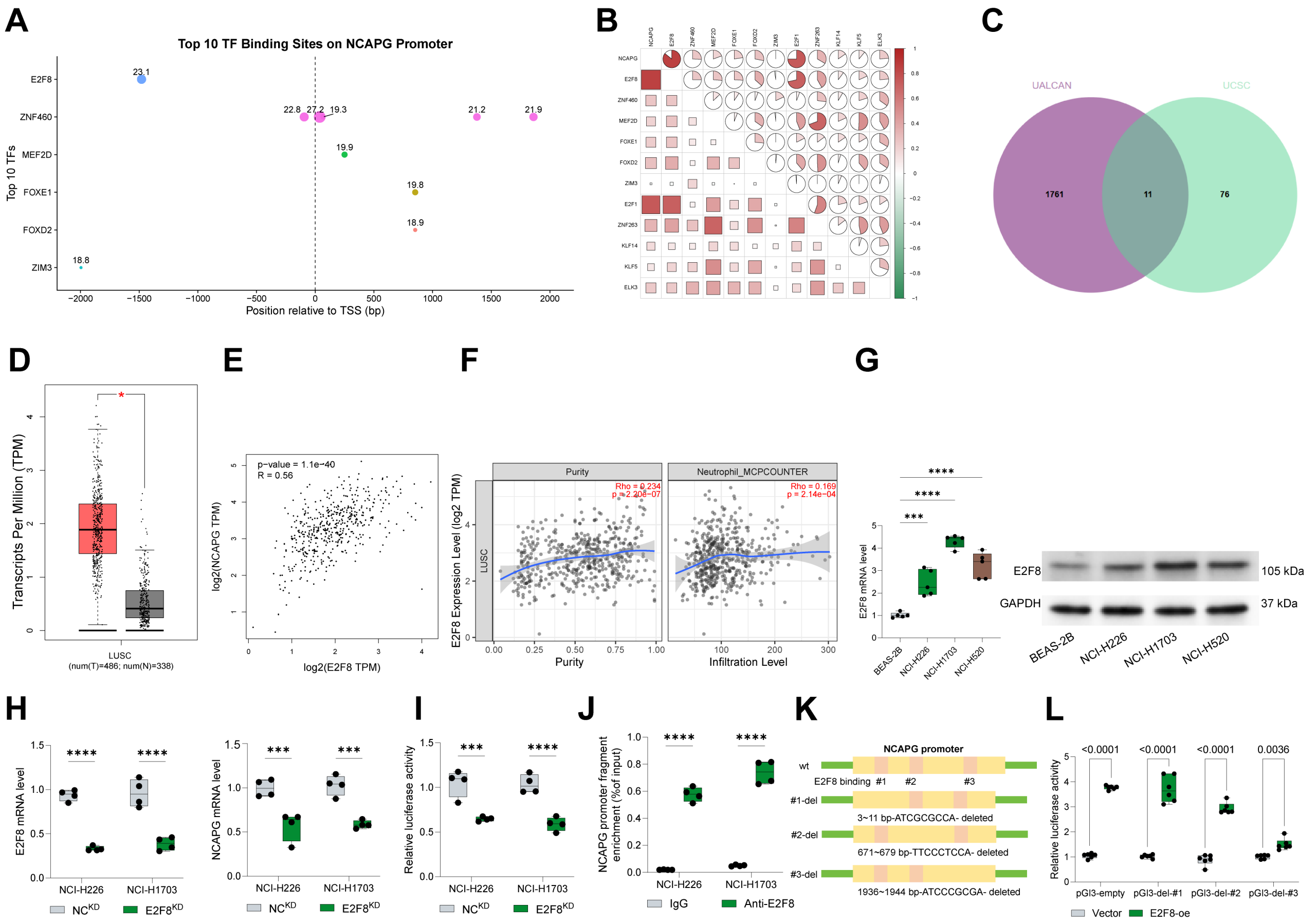

Fig. S3. The transcription factor binding to NCAPG promoter. (A) Transcription factors binding to the NCAPG promoter region (chr4:17810655-17811004) predicted using the UCSC database. (B) Heatmap for NCAPG-associated genes in LUSC generated using the UALCAN system. (C) Intersections of TFs binding to the NCAPG promoter and NCAPG-associated genes. Expression pattern of E2F8 (D) and its correlation with NCAPG expression (E) in LUSC predicted using the GEPIA system. (F) Correlation between E2F8 expression and neutrophil infiltration in LUSC predicted using the Sangerbox tool. (G) Expression levels of E2F8 in LUSC cell lines (NCI-H226 and NCI-H1703) and normal BEAS-2B cells measured using RT-qPCR and WB analysis. (H) mRNA expression of E2F8 and NCAPG in NCI-H226 and NCI-H1703 cells after administration of E2F8^{KD} or NC^{KD} measured using RT-qPCR. (I) Regulation of E2F8 on NCAPG determined using luciferase

assay. (J) Binding of E2F8 to the NCAPG promoter analyzed using ChIP-qPCR assay. (K-L) Constructed pGL3-promoter luciferase reporter vectors containing the wild-type (wt) sequence, as well as deletion mutants for each binding site: del-#1 (binding site 1 deleted), del-#2 (binding site 2 deleted), and del-#3 (binding site 3 deleted). These reporter constructs were then co-transfected with E2F8 into 293T cells. Data are expressed as the mean ± SD and shown as dot plots with whiskers representing SD. In vitro assays represent n = 6 independent biological replicates. For animal experiments, n = 6 biologically independent mice were analyzed per group. Data are presented as dot-and-whisker plots. ***$p < 0.001$, ****$p < 0.0001$.

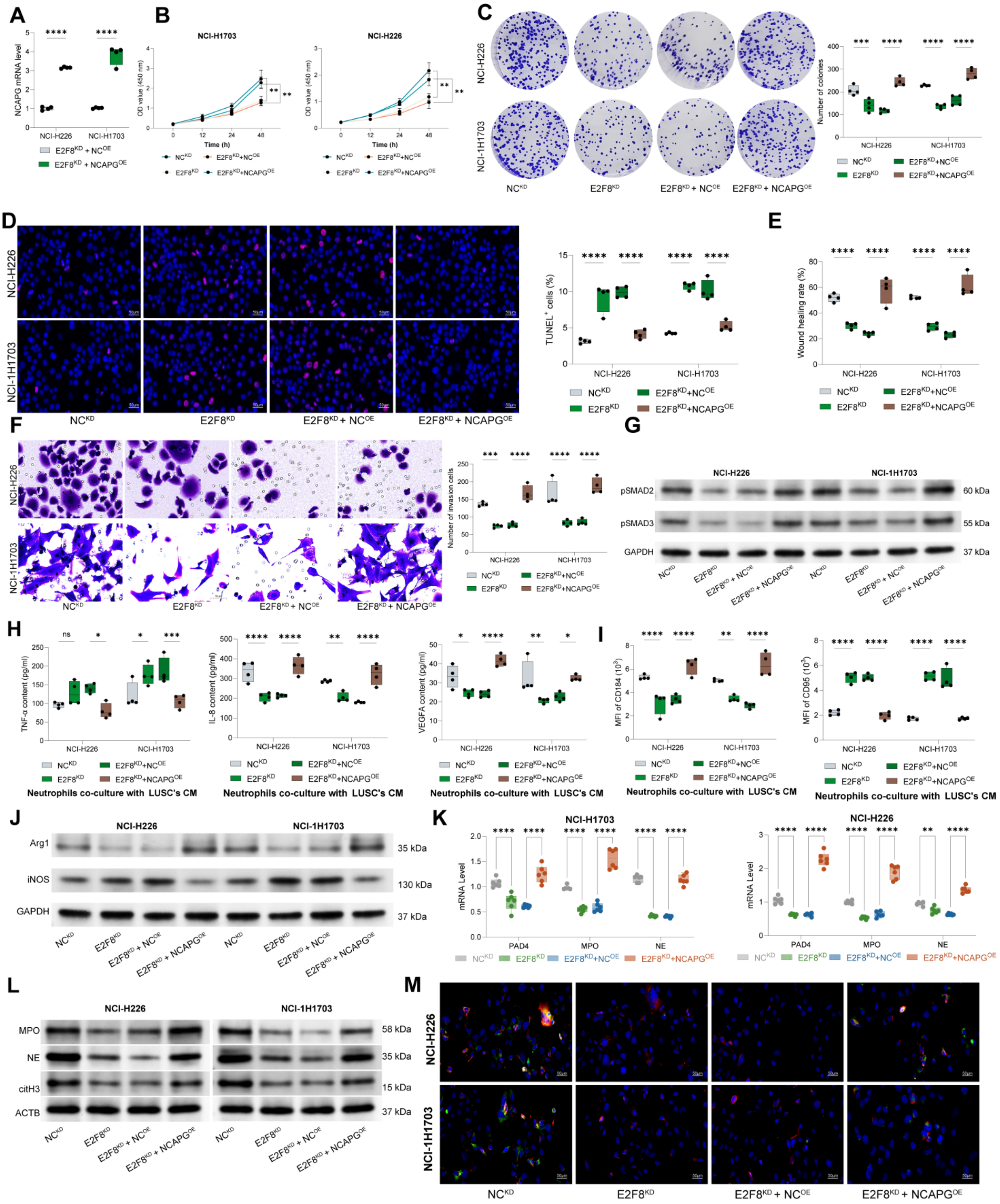

Fig. S4 NCAPG overexpression restores malignant behavior of LUSC cells upon E2F8 knockdown. NCI-H226 and NCI-H1703 cells were transfected with E2F8^{KD} alone or along with NCAPG^{OE}. (A) mRNA expression of NCAPG in NCI-H226 and NCI-H1703 cells after transfection determined using RT-qPCR. (B) Proliferation of cells after transfection analyzed by CCK-8 assays. (C) Colony formation ability of cells after transfection. (D) Apoptosis in transfected cells measured using TUNEL

assay. (E) Migration of cells assessed using wound healing assay. (F) Invasion of cells assessed using Transwell assay. Each dot indicates an independent experiment. (G) Levels of p-SMAD2 and p-SMAD3 in LUSC cells with E2F8 knockdown and NCAPG overexpression determined using WB analysis. Neutrophils were cultured in CM derived from the differentially transfected LUSC cells. (H) Concentrations of IL-8, VEGFA, and TNF-α in the cell culture supernatant determined using ELISA kits. (I) Expression of TAN N1 marker CD95 and N2 marker CD184 in neutrophils after culture in the CM analyzed using flow cytometry. (J) Protein levels of Arg-1 and iNOS in neutrophils after culture in the CM determined using WB analysis. (K) mRNA expression of MPO, NE, and PAD4 in neutrophils after culture in the CM analyzed using qPCR analysis (L) Protein levels of MPO, NE, and cit-H3 in neutrophils after culture in the CM analyzed using WB analysis. (M) Positive staining of MPO and cit-H3 in neutrophils after culture in the CM determined using immunofluorescence staining. Data are expressed as the mean ± SD and shown as dot plots with whiskers representing SD. In vitro assays represent n = 6 independent biological replicates. For animal experiments, n = 6 biologically independent mice were analyzed per group. Data are presented as dot-and-whisker plots. $^{**}p < 0.01$, $^{***}p < 0.001$, $^{****}p < 0.0001$.

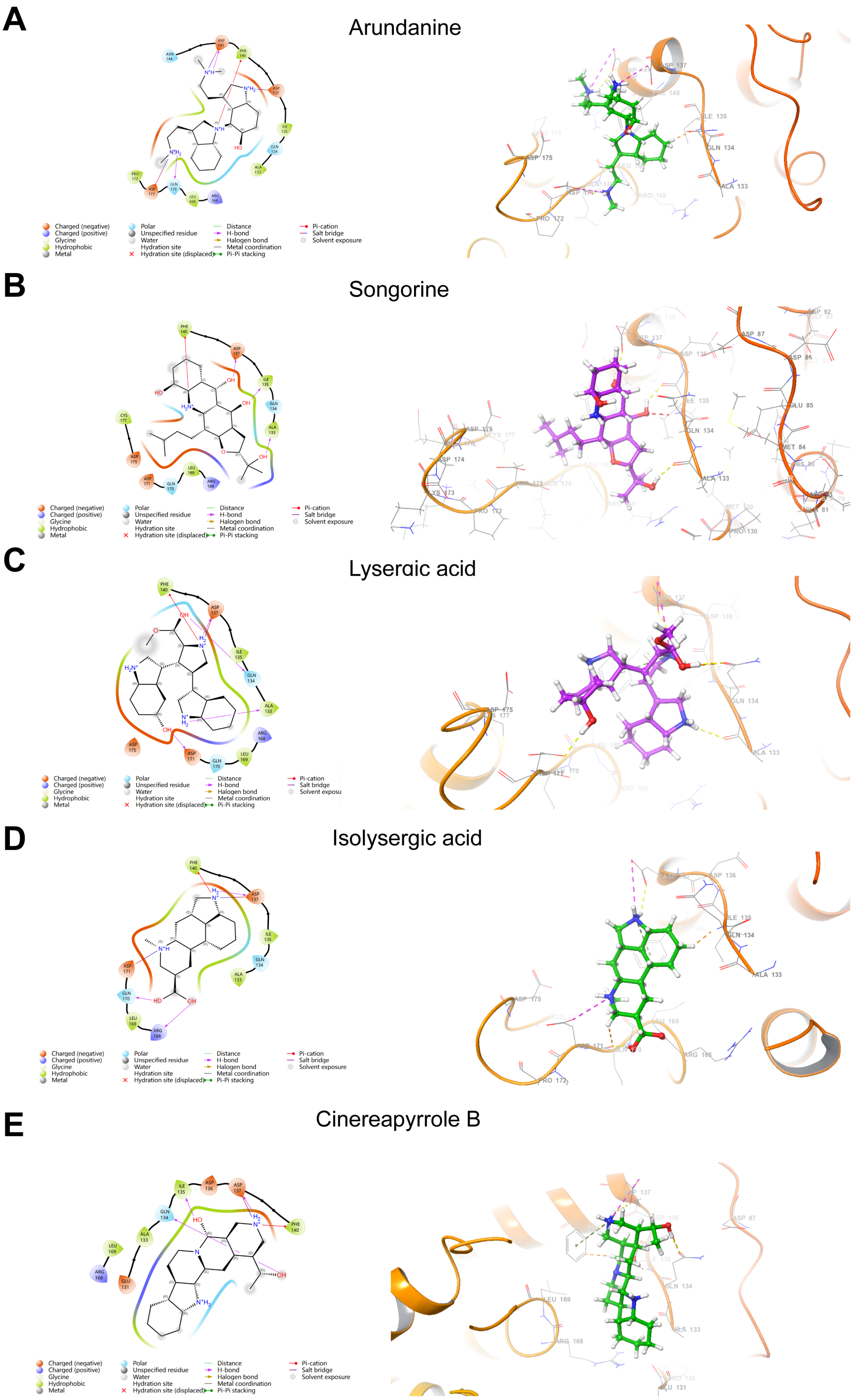

Fig. S5. Vina docking analysis of natural compounds capable of binding to NCAPG. (A-E) Arundanine, Songorine, Lysergic acid, Isolysergic acid, and Cinereapyrrole B, respectively.

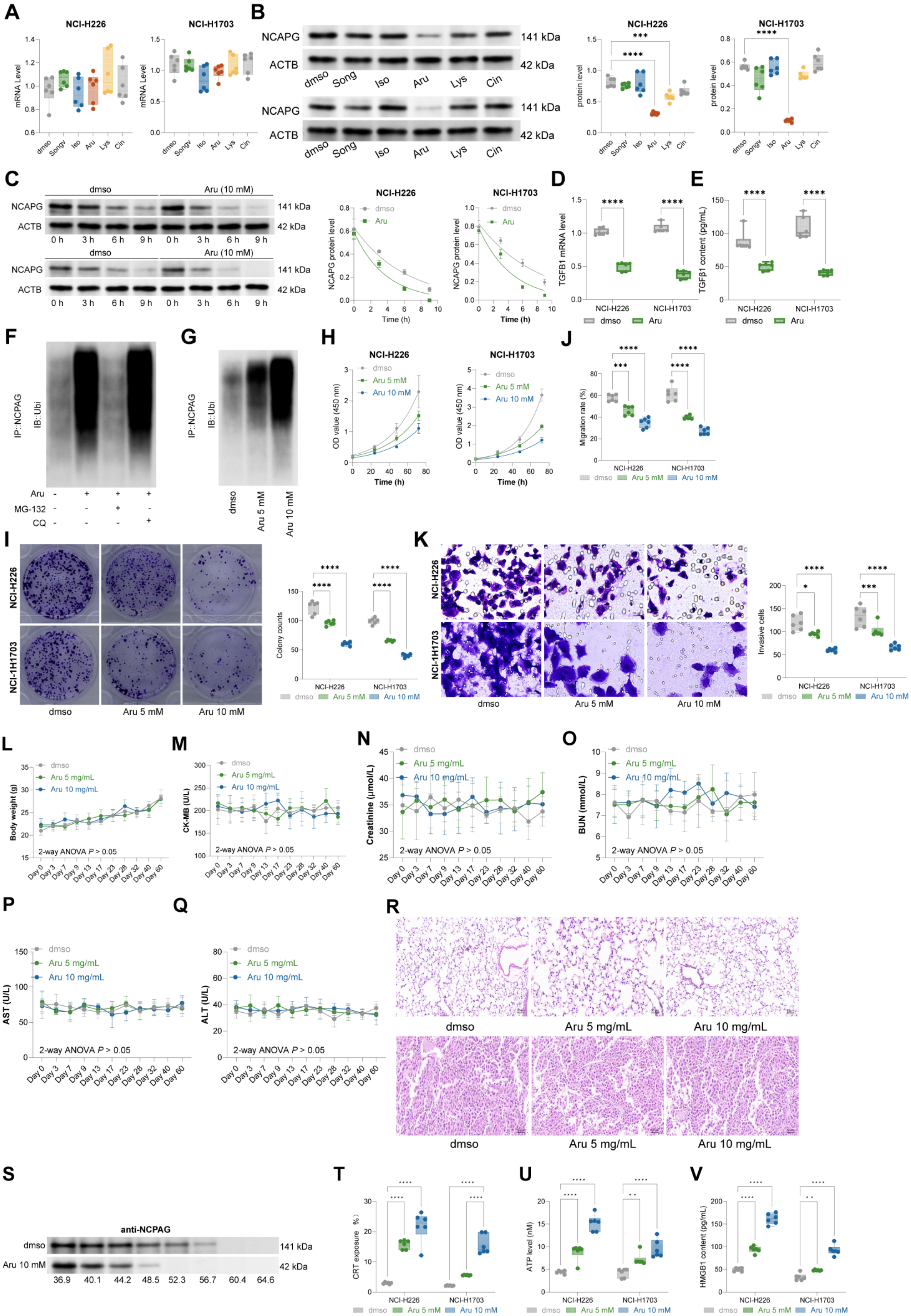

A
NCI-H226
NCI-H1703
mRNA Level
dmso
Songv
Iso
Aru
Lys
Cin
B
NCAPG
ACTB
141 kDa
42 kDa
dmso
Song
Iso
Aru
Lys
Cin
NCI-H226
NCI-H1703
protein level
C
dmso
Aru (10 mM)
NCAPG
ACTB
141 kDa
42 kDa
0 h
3 h
6 h
9 h
NCI-H226
NCI-H1703
NCAPG protein level
Time (h)
D
TGFB1 mRNA level
E
TGFβ1 content (pg/mL)
F
IP::NCPAG
IB::Ubi
Aru
MG-132
CQ
G
Aru 5 mM
Aru 10 mM
H
OD value (450 nm)
Time (h)
J
Migration rate (%)
I
NCI-H226
NCI-1H1703
Colony counts
K
Invasive cells
L
Body weight (g)
2-way ANOVA P > 0.05
Aru 5 mg/mL
Aru 10 mg/mL
M
CK-MB (U/L)
N
Creatinine (μmol/L)
O
BUN (mmol/L)
Day 0
Day 3
Day 7
Day 9
Day 13
Day 17
Day 23
Day 28
Day 32
Day 40
Day 60
P
AST (U/L)
Q
ALT (U/L)
R
S
anti-NCPAG
dmso
Aru 10 mM
141 kDa
42 kDa
36.9
40.1
44.2
48.5
52.3
56.7
60.4
64.6
T
CRT exposure (%)
U
ATP level (nM)
V
HMGB1 content (pg/mL)

Fig. S6. Arundanine inhibits NCAPG activity in LUSC cells. NCI-H226 and NCI-H1703 cells were treated with the IC_{50} values of Arundanine, Songorine, Isolysergic acid, Lysergic acid, or Cinereapyrrole B. (A-B) qPCR (A) and WB analysis (B) to detect NCAPG mRNA and protein expression levels in cells across groups. (C) NCI-H226 and NCI-H1703 cells were treated with the protein synthesis inhibitor cycloheximide and then with Arundanine (5 mM or 10 mM), followed by WB analysis to determine NCAPG protein stability. (D-E) NCI-H226 and NCI-H1703 cells were treated with gradient concentrations of Arundanine, followed by qPCR and ELISA to detect TGFB1 mRNA and TGF-β1 secretion levels. (F) Evaluation of NCAPG protein expression in Arundanine-treated cells co-incubated with the proteasome inhibitor MG132 or the lysosomal inhibitor Chloroquine (CQ) to delineate the specific degradation pathway. (G) Immunoprecipitation (IP) assay demonstrating the polyubiquitination levels of NCAPG following treatment with Arundanine. (H) Proliferation of cells after Arundanine treatment analyzed by CCK-8 assays. (I) Colony formation ability of cells after Arundanine treatment. (J) Migration of cells assessed using wound healing assay. (K) Invasion of cells assessed using Transwell assay. (L) Body weight monitoring of mice treated with vehicle (DMSO), 5 mg/kg Arundanine, or 10 mg/kg Arundanine over a 60-day period. (M-Q) Serum biochemical analysis was performed to assess potential organ toxicity. The levels of (M) CK-MB (heart), (N) Creatinine and (O) BUN (kidney), as well as (P) AST and (Q) ALT (liver) were measured at the indicated time points. (R) Representative Hematoxylin and Eosin (H&E) staining images of lung (top row) and liver (bottom row) tissues collected at the endpoint. (S) Cellular Thermal Shift Assay (CETSA) demonstrating the ligand-induced thermal stabilization of NCAPG in intact LUSC cells following Arundanine treatment, confirming a direct physical interaction. (T) Flow cytometric quantification of cell-surface calreticulin (CRT) exposure on NCI-H226 and NCI-H1703 cells treated with Arundanine to assess the induction of Immunogenic Cell Death (ICD). (U-V) Evaluation of extracellular DAMP release, demonstrating the dose-dependent accumulation of ATP (U) and HMGB1 (V) in the culture supernatant following Arundanine treatment. Data are expressed as the mean ± SD and shown as dot plots with whiskers representing SD. In vitro assays represent n

= 6 independent biological replicates. For animal experiments, n = 6 biologically independent mice were analyzed per group. $^{**}p < 0.001$, $^{***}p < 0.001$, $^{****}p < 0.0001$.

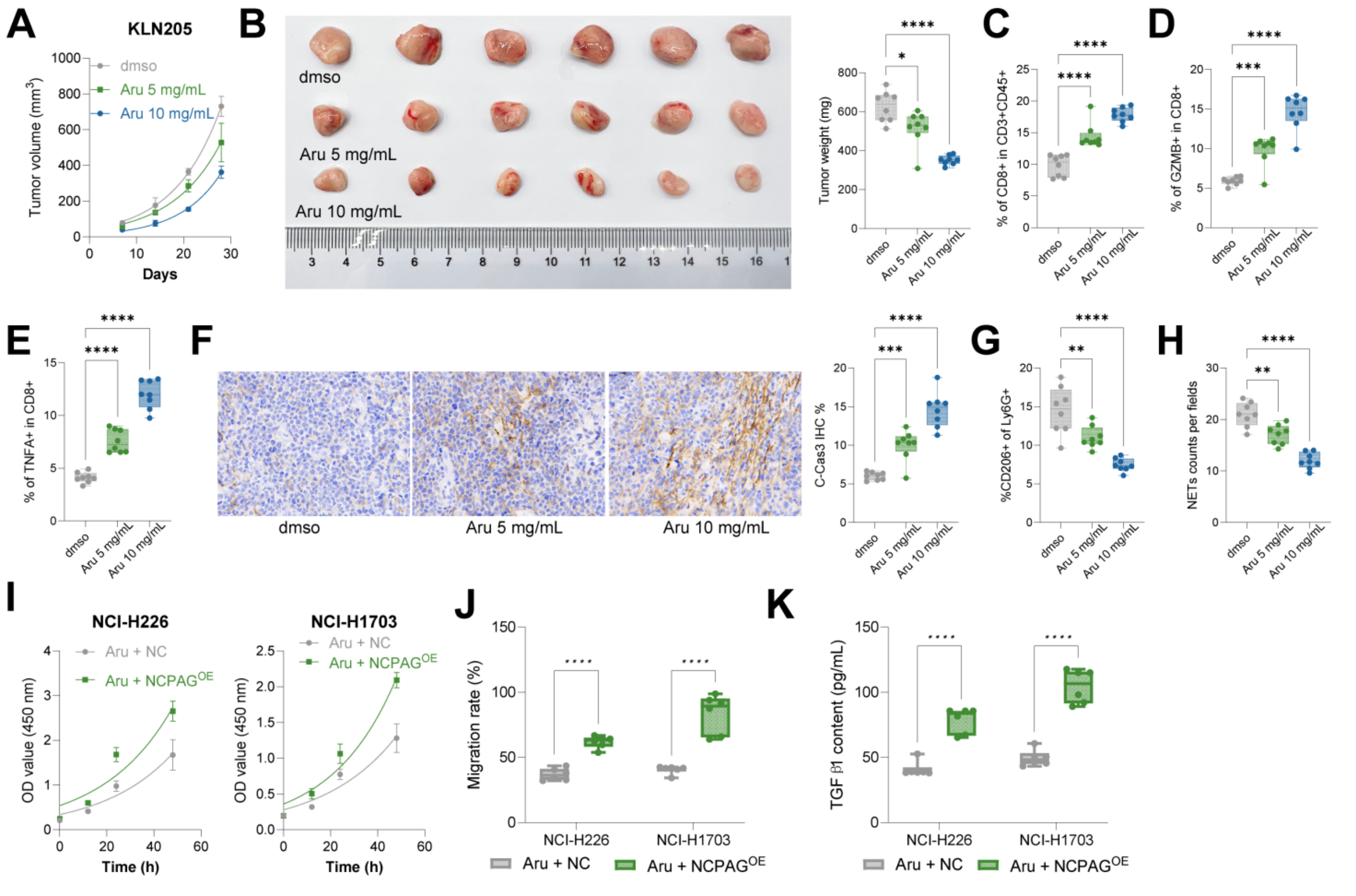

Fig. S7. Arundanine restricts NET formation and enhances the cytotoxic effect of $CD8^+$ T cells. KLN205 cells were inoculated into DBA/2 mice, followed by Arundanine treatment (5 mg/kg or 10 mg/kg) starting from day 8. (A) Growth rate of subcutaneous tumors in mice over 28 days (B) Representative images and weights of subcutaneous tumors in mice on day 28 (C-E) Flow cytometry detection of the number of $CD3^+CD45^+CD8^+$ (C), $CD8^+TNF\text{-}\alpha^+$ (D), and $CD8^+GZMB^+$ (E) T cells in tumor tissues. (F) IHC detection of staining intensity for cleaved caspase-3 in tumor tissues. (G) Flow cytometry detection of the number of $Ly6G^+CD206^+$ cells in tumor tissues. (H) NET formation determined using immunofluorescence staining of MPO and cit-H3. (I-K) Evaluation of functional rescue in NCI-H226 and NCI-H1703 cells stably overexpressing NCAPG ($NCAPG^{OE}$) compared to vector controls following treatment with 10 mg/kg Arundanine. The panels demonstrate the abrogation of Arundanine-induced suppressive effects on cell viability (I), cellular migration capacity (J), and TGF-β1 secretion (K). Data are expressed as the mean ± SD and shown as dot plots with

whiskers representing SD. In vitro assays represent n = 6 independent biological replicates. For animal experiments, n = 6 biologically independent mice were analyzed per group. Data are presented as dot-and-whisker plots. *$p < 0.05$, **$p < 0.01$, ***$p < 0.001$, ****$p < 0.0001$.

Supplementary Table 1

TPX2	CKS1B	ASPM	MKI67
DEPDC1B	SNRPE	FAM83B	FERMT1
SERPINB5	TMEM14A	MND1	HMMR
DSP	KIF4A	PRC1	CCT5
NOSTRIN	ABI3BP	CENPW	KRT5
GINS1	TYMS	CKAP2	KRT6B
ACTL6A	MELK	CEP55	GPR87
TIMP3	NDC80	KRT16	TTK
HIGD1B	RFC4	TOP2A	NCAPG
ANLN	NUF2	LDB2	CCDC58
BIRC5	KRT6A	TUBA1C	NEK2
CHEK1	PTTG1	CDC45	TP63
KIF11	UBE2T	NEXN	CENPF
CDC6	ECT2	S100A2	NUSAP1
DSG2	KIF23	SPC25	
TNNC1	ADGRF5	DSG3	
AURKA	EMCN	BUB1B	
NDNF	CGNL1	DLGAP5	
PERP	DSC3	RAD51AP1	
KRT17	CENPE	FMO2	
